# Adapting a Self-Guided eHealth Intervention Into a Tailored Therapist-Guided eHealth Intervention for Survivors of Colorectal Cancer

**DOI:** 10.2196/63486

**Published:** 2025-03-05

**Authors:** Johanne Dam Lyhne, Allan ‘Ben’ Smith, Tina Birgitte Wisbech Carstensen, Lisa Beatty, Adeola Bamgboje-Ayodele, Britt Klein, Lars Henrik Jensen, Lisbeth Frostholm

**Affiliations:** 1 Department of Clinical Oncology, University Hospital of Southern Denmark, Beriderbakken 4, Vejle, 7100, Denmark, 45 24453561; 2 Daffodil Centre, A joint venture between Cancer Council NSW and University of Sydney, Sydney, Australia; 3 Clinic for Functional Disorders, Aarhus University Hospital, Aarhus, Denmark; 4 Department of Clinical Medicine, Aarhus University, Aarhus, Denmark; 5 Flinders University Institute of Mental Health & Wellbeing, College of Education, Psychology & Social Work, Flinders University, Adelaide, Australia; 6 Psycho-Oncology Cooperative Research Group, Sydney, Australia; 7 Ingham Institute for Applied Medical Research, South West Sydney Clinical Campuses, UNSW Medicine & Health, University of New South Wales, Liverpool, New South Wales, Australia; 8 Biomedical Informatics and Digital Health, School of Medical Sciences, Charles Perkins Centre, Faculty of Medicine and Health, University of Sydney, Sydney, Australia; 9 Health Innovation & Transformation Centre, Federation University Australia, Ballarat, Australia; 10 Biopsychosocial and eHealth Research & Innovation Hub, Federation University Australia, Ballarat, Australia

**Keywords:** fear of cancer recurrence, therapist-guided, self-guided, online intervention, colorectal cancer, digital health, psychosocial intervention, survivorship, eHealth, adaptation, survivors, oncologists, therapists, acceptability, mobile phone

## Abstract

Therapist-guided eHealth interventions have been shown to engage users more effectively and achieve better outcomes than self-guided interventions when addressing psychological symptoms. Building on this evidence, this viewpoint aimed to describe the adaptation of iConquerFear, a self-guided eHealth intervention targeting fear of cancer recurrence, into a therapist-guided version (TG-iConquerFear) tailored specifically for survivors of colorectal cancer (CRC). The goal was to optimize patient outcomes while minimizing the need for extensive resources. The adaptation process followed the Information System research framework, which facilitated a systematic integration of knowledge and iterative testing. Drawing on insights from the original iConquerFear development, as well as feedback from end users, oncologists, and therapists, we began by identifying areas for improvement. These insights formed the foundation for the first design cycle. Initial internal testing revealed the need for several adjustments to enhance the intervention. While the core concept of iConquerFear remained unchanged, we made significant modifications to improve access by optimizing the platform for mobile devices, to support adherence by expanding the exercises, and to equip therapists with tools such as reflective questions and a monitoring control panel. External field testing with 5 survivors of CRC provided further validation. Participants reported a high level of acceptability, and their feedback guided additional minor points to consider incorporating in future versions. This study illustrates how a self-guided eHealth intervention can be successfully adapted into a therapist-guided format for fear of cancer recurrence, tailored to meet the needs of survivors of CRC. The described approach serves as a valuable framework for integrating therapist guidance into similar interventions, ensuring their relevance and effectiveness for targeted populations.

## Introduction

eHealth interventions, defined as programs that provide information and support for physical or mental health problems via digital platforms [1], can overcome barriers to accessing support including travelling distance to site of intervention, time constraints, disease burden, financial issues, perceived stigma, and mobility or logistics constraints due to pandemics such as COVID-19 [2]. eHealth interventions in diverse cancer settings address challenges related to scalability and cost-effectiveness with effects comparable to traditional face-to-face therapy [3-7]. However, the initial development, evaluation, and implementation of effective eHealth interventions is a complex and multidisciplinary process [8], which requires substantive financial and human resources [9].

eHealth interventions may fill an important gap in psychosocial cancer care especially, by augmenting limited available services [10]. However, the efficacy of self-guided psychological eHealth interventions can be limited by low uptake and engagement [11]. Furthermore, these interventions might increase disparities in health care, as those with digital skills and more resources will be more likely to engage [12], while those with late effects such as peripheral neuropathy and fatigue might face challenges in using required devices (eg, computers, keyboards, or mice) [13]. Promisingly, meta-analytic evidence shows that adding guidance to interventions yields greater efficacy when treating anxiety, distress, fatigue [14], and fear of cancer recurrence (FCR) [7] in people living with cancer compared with nonguided interventions. However, guidance comes with greater costs and reduced scalability due to the use of health care personnel, infrastructure, and safety measures [15-19].

Leveraging existing self-guided eHealth interventions is one way of reducing resources required to design an entirely new guided eHealth service. Several psycho-oncological interventions have undergone successful adaptations, for example FindingMyWay [20] from Australia and Fear Of Recurrence Therapy [21] from Canada. FindingMyWay has undergone 2 adaptations: first, into a UK-version needing a contextual adaptation [22] to reflect the UK health care system and terminology, and second, to a metastatic-breast cancer specific version (Finding My Way-Advanced [23]). Fear Of Recurrence Therapy was adapted to family caregivers and to an eHealth format [24].

Intervention models that facilitate greater access to FCR treatment have been identified as a top international research priority [25]. To address this priority, Smith et al [26] adapted the effective face-to-face therapist-delivered ConquerFear [27] FCR treatment to an eHealth self-guided format (iConquerFear). However, feasibility testing of iConquerFear revealed that some individuals needed guidance and more relatable content for optimal engagement and benefit [28], as reported for other psychological symptoms [15,29]. To address these two key recommendations, this current study aimed to adapt iConquerFear into a tailored asynchronous therapist-guided eHealth version (TG-iConquerFear).

While guidance can be provided either in real time (synchronous) or as delayed (asynchronous), a systematic review by Cox et al [30] has shown the superior convenience, flexibility, and limited interruptions of daily routines with asynchronous guidance in telehealth interventions. As recommendations are mixed [31,32] the choice of asynchronous guidance will be evaluated.

To make intervention content more personal and relatable, a further aim of this study was to tailor iConquerFear specifically for survivors of colorectal cancer (CRC). CRC ranks as the third most frequently diagnosed cancer worldwide, with its prevalence steadily rising due to prolonged survivorship [33]. The prevalence and characteristics of FCR experienced by individuals affected by CRC have been described in detail [34-40]. However, no intervention customized to address FCR in survivors of CRC has been developed [7,41].

In summary, this paper describes the process of adapting iConquerFear into TG-iConquerFear targeting survivors of CRC. We report using the Information System research framework [42] to integrate recommended improvements from the original Australian development study [26] and pilot study [28] with end user feedback from field testing with oversight by a multidisciplinary research team as a template for other researchers seeking to make similar adaptations.

## Methods

### Intervention Content

The iConquerFear is a metacognitive intervention consisting of 5 modules. The content of each module is outlined in Table 1 [26].

**Table 1.** iConquerFear content and features. Features common across all modules include the following: web-based questionnaires, interactive exercises, downloadable hand-outs, progress graphs, email and SMS reminders, and safety plan.

Module	Content and features
1. Introduction and goal setting	Introduction to FCR^a^ and treatment model (survivor and clinician videos) Values clarification and goal setting (interactive card sort exercise)
2. Attention training	Introduction to attention training (survivor and clinician videos) Attention training practice (audio, monitoring and feedback, and reminders)
3. Detached mindfulness	Introduction to detached mindfulness (survivor and clinician videos) Detached mindfulness practice (audio, monitoring and feedback, and reminders)
4. Learning to live well and manage worry	Psychoeducation about appropriate threat monitoring (clinician video) Compliance with follow-up and self-examination (assessment with feedback) Challenging unhelpful metacognition (assessment with feedback) Worry management techniques (textual overview and downloadable PDF)
5. Treatment summery and relapse plan	Reflection on change in FCR during treatment (assessment with feedback) Consolidation of new strategies for managing FCR through relapse prevention (personalized feedback and downloadable action plan)

a FCR: fear of cancer recurrence.

### Language and Cultural Adaptation

The adaptation of iConquerFear to TG-iConquerFear was preceded by a draft translation and cultural adaptation of all written material from iConquerFear by a professional translator with experience in the psychiatric setting (Multimedia Appendix 1). This Danish draft was then built into an existing web-based treatment platform made available by the Department for Functional Disorders at Aarhus University Hospital in Denmark. The design of the Danish platform was comparable to that of iConquerFear. When needed, new technical features were programmed to reflect the intervention treatment content of iConquerFear.

### Framework

**Figure 1.**
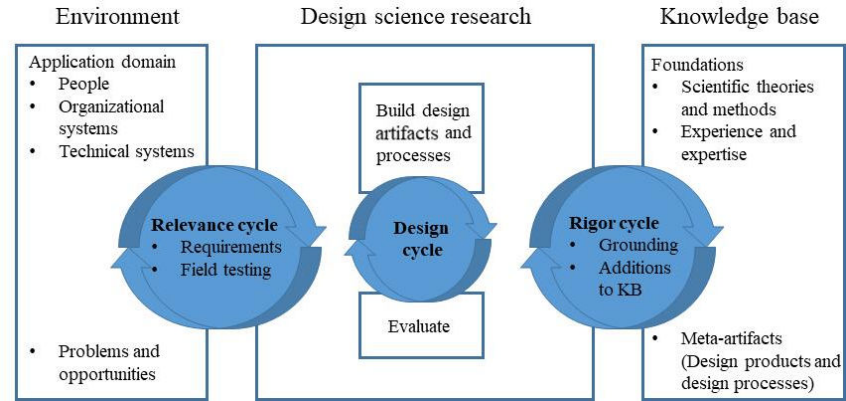
The Information System research framework. KB: knowledge base.

A participatory design approach, guided by the Information System research framework [42], was used in the adaptation process. The Information System research framework urges end users’ inclusion and active engagement in designing and evaluating information systems. The framework consists of 3 overarching user participatory design cycles: The relevance cycle determines end user requirements; the design cycle involves prototype development and evaluation; the rigor cycle focuses on assessing “past knowledge” from the knowledge base (KB) and underpinning theories (Figure 1) [42].

### Adaptation Process

At each stage of adaptation, the intervention was reviewed by a multidisciplinary research team (8 members), comprised of: an oncologist specializing in CRC, a health psychologist with expertise in developing guided online interventions, one of the iConquerFear developers, 2 psychologists specializing in anxiety-related online therapy (the therapists), a health researcher with expertise in psycho-oncology, and 2 survivors of CRC.

#### Relevance Cycle

First, information related to the adapted intervention’s relevance were gathered from the environment. Three consumer representatives (community partners from the Danish Bowel Cancer Association and the Association for Late Effects After Cancer), and 4 people with lived experience (survivors of CRC) were consulted for problem definition and justification of the interventions’ relevance, including need and potential challenges. Within the same two 1-hour focus groups, the main elements from the iConquerFear intervention were presented to explore views on whether the online platform would be relevant in addressing survivors of CRC needs and how to promote engagement. The challenges raised in these interviews were validated with existing literature on online intervention engagement. The environment was revisited twice during internal and external field testing.

#### Rigor Cycle

Existing knowledge from the KB consisted mainly of expertise and experience from the development [26] and piloting of iConquerFear [28], which was rigorously developed based on the ConquerFear therapy manual [43] and iterative user feedback. This knowledge was complemented by feedback on the iConquerFear program from the TG-iConquerFear research team. Research team members evaluated iConquerFear content to assess suitability for survivors of CRC and the therapist-guided format. Feedback was collected via email or shared during live meetings. A selected group of research team members reviewed all aspects of the modules, including exercises, text, images, graphics, examples, and videos. Group discussions were held to assess their relevance to survivors of CRC experiencing elevated FCR. The unique needs and challenges specific for survivors of CRC were also deliberated, playing a crucial role in making intervention content more personal and relatable. The core therapeutic concept of iConquerFear, including general structure, therapeutic principles, and key goals remained unaltered in TG-iConquerFear.

#### Design Cycle

Insights added through the relevance cycle and rigor cycle contributed to the design science research consisting of implementation of all knowledge and suggestions into iConquerFear. The loop in the design cycle was between members of the research team and 3 software designers. Multiple iterations of the design cycle were conducted before internally field-testing TG-iConquerFear version 1 in the environment as part of a further relevance cycle. The 3 cycles allowed for an iterative process resulting in a second version, which was field-tested externally.

#### Field Testing

During both internal and external field testing, the intervention was used as intended based on the original manual from PoCoG (Psycho-Oncology Co-Operative Research Group) in Australia, with therapist guidance provided over a span of 10 weeks. The amount of therapist-guidance was measured by assessing the quality, quantity and length of messages exchanged between therapist and participant in each module. Every participant had a designated personal therapist and messages were answered within 3 working days. As the main focus of the guidance was to increase engagement and adherence, the substance of the guidance was not predetermined or restricted, and no limits were established concerning frequency of communication. It was planned that there would be communication between participant and therapist at least once a week. Both therapist and participant could initiate communication. Specific tasks for the therapist included welcoming the participant, addressing inquiries regarding content and tools, providing feedback on exercises within the program, and motivating engagement and adherence.

#### Internal Field Testing

The internal field testing was performed within the research team. The 2 survivors of CRC were test-participants and the 2 therapists, who have extensive experience in online therapy, provided the guidance. Both the survivors of CRC and the therapists gave written feedback on various feasibility measures, including usability, adherence, acceptability, and safety after each module and at the end of the intervention.

#### External Field Testing

For the external field testing, interested volunteers from the bowel cancer association were informed about the project by telephone by the primary investigator and subsequently screened for eligibility via an online questionnaire sent directly to their digital citizen mailbox (used for communication between citizens and public authorities in Denmark). Volunteers were eligible if they were aged 18 years or older, had completed surgery for CRC with curative intent, were without sign of recurrence, and gave electronic informed consent. A minimum Fear of Cancer Recurrence Inventory–Short Form score of 13, indicating moderate or higher FCR levels [44], was also required. Volunteers were excluded if they self-reported clinical levels of depression, psychotic illness, or abuse of alcohol or drugs. Volunteer participants were given a unique link to the online platform of TG-iConquerFear. The guidance was performed by the same 2 therapists from the research team. Participants and therapists were encouraged to provide written feedback to the primary investigator on usefulness and suggestions for improvement after completing each module, and at the end of the intervention. This study was approved by the Regional Research Ethics Committee of Southern Denmark (S—20190061).

## Results

### TG-iConquerFear Version 1

**Figure 2.**
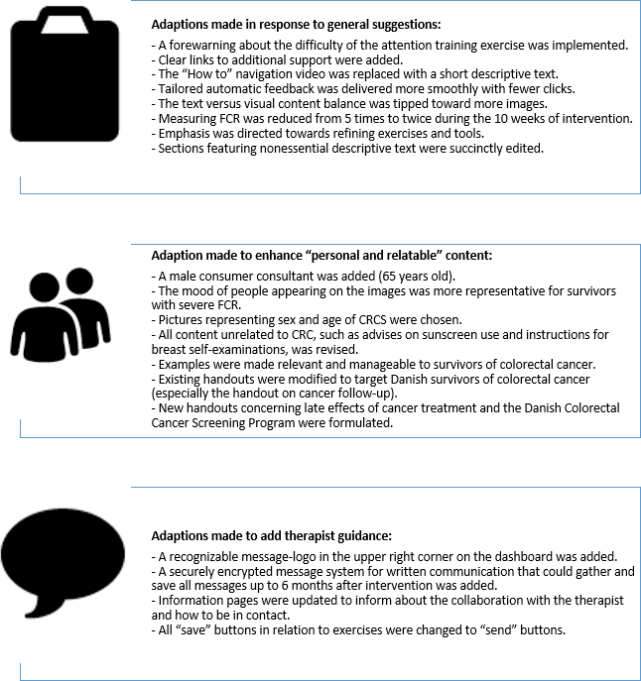
Specific adaptations made in response to suggestions from the environment and the knowledge base. CRC: colorectal cancer; FCR: fear of cancer recurrence.

During the initial relevance cycle, group interviews with representatives from the environment (consumer representatives and survivors of CRC) justified adapting iConquerFear to better engage prospective participants. Identified challenges included concerns about the complexity of pages overloaded with text (“You need to get to the point faster”), relevance of certain elements (such as sunscreen and breast palpation; “that just annoys me”), videos featuring only women (“seems like something only women have?”), and assessing further support if needed. “… Being on your own” was a major concern, and given that a large portion of end users are expected to be used, the focus group participants favored asynchronous communication. Suggestions for adaptations from the KB were either general or focused on 2 key recommendations: enhancing personal and relatable content, and adding guidance. Suggestions for adaptations from the research team centered on how to integrate therapist guidance, drawing from experience with eHealth interventions targeting health anxiety, and how to tailor hand-outs to address the specific needs of survivors of CRC, particularly regarding follow-up and late effects. These suggestions served as guidance for the design cycle, during which the adapted TG-iConquerFear version 1 was crafted. The specific adaptations made in response to these suggestions are outlined in Figure 2.

TG-iConquerFear version 1 then progressed to the relevance cycle, where it underwent internal field testing in the environment.

### Internal Field Testing

The outcomes from the internal field testing are presented in Table 2. The feedback was presented to the research team, who determined that a second design cycle was necessary. Adjustments were made to TG-iConquerFear version 1 (last column in Table 2), leading to TG-iConquerFear version 2.

**Table 2.** Outcomes from internal field testing and adjustments.

Feasibility measure and feedback from end users		Adjustments
Usability		
	“It has proven to be a barrier for me that the system cannot be accessed via a smartphone. In an already busy daily life, I repeatedly find that I do not sit down at my PC.” [Female participant, aged 51 years]	The intervention was adjusted to enable easier access on smart phone and iPad.
	“It takes really long time to figure out how far the participants are within the program, and if they had made any new exercises since last therapist log-in.” [Female therapist, aged 38 years]	A therapist-monitored control panel with information on all active participants within treatment, including registration of activity for each participant, and expected date for completion of the program was added.
Usability of the asynchronous communication		
	“In many ways, it’s easier to sit here and write with you than if we were face to face - I like that I have the opportunity to write something, consider, rephrase, etc - or just write freely in a flood of ‘unloading,’ depending on what I need on that day.” [Female participant, aged 51 years]	No adjustments
	“For me as a therapist, asynchronous or delayed communication between me and the patient, means that I don’t have to schedule specific agreed-upon sessions with patients… Additionally, I can take my time to consider the responses I give to patients. Sometimes, in face-to-face interactions, it can be challenging to find the right words or consider the right way to challenge the patient in the moment. Asynchronous communication with the patients makes my work less stressful and more flexible.” [Female therapist, aged 48 years]	No adjustments
Adherence		
	“We need a clear indication from the participants on whether or not they have been working with the tools and find them useful.” [Female therapist, aged 38 years]	In each module, two reflective questions with tailored feedback were added to monitor if the participants had engaged with the exercises of the previous module, and if and how the exercises had been helpful.
Acceptability		
	“The value-based module is too short for 14 days of training, and it is not clear how the participant should work with specific goals that are in line with their values. This results in a rather quick completion of the module and the participants state rather vague and abstract goals without a clear indication of when reaching the goal.” [Female therapist, aged 48 years]	A lighthouse metaphor was added to explain values, see Multimedia Appendix 2, for the full formulation.
	"I find especially the first exercises (ref: the value clarification exercise) inadequately explained. For academics with a background in social sciences, the explanations are not so difficult to interpret, but they probably make up the smallest part of the target audience, which is likely more composed of older individuals from all social strata. Among them, there may be many who have difficulty benefiting from this module if they are not further guided.” [Male participant, aged 67 years]	Information on the value-clarification exercise was made more concrete so that the program itself was able to guide the participant through the exercises leading the therapist to stand back and support and evaluate.
Safety		
	“I hesitated to fully engage in this digital treatment. I needed my therapist to ensure confidentiality.” [Female participant, aged 51 years]	A paragraph on confidentiality was added in the introduction.

### TG-iConquerFear Version 2

Following the adjustments from the first relevance cycle, the second design cycle aimed to refine TG-iConquerFear version 1 further. Key modifications, as detailed in Table 2, were implemented to address user feedback and enhance the intervention’s feasibility. This second version underwent external field testing within the environment.

### External Field Testing

The external field testing took place from February to July 2022 at the Clinic for Functional Disorders at Aarhus University Hospital in Denmark. It involved 5 volunteers from the Danish Bowel Cancer Association. All 5 were survivors of colon cancer and most were female (n=4), married (n=4), and employed (n=4). Mean age was 54 (SD 10.2; range 42-71) years and average time since diagnosis were 2.3 (SD 1.6) years (8 months to 5 years).

Four test-participants completed all 5 modules, while 1 test-participant did not complete module 2. The amount of therapist guidance averaged 15.2 (range 12‐23) messages per participant, equating to 2.7 (range 2‐3.8) messages per module. The mean length of a message was 196.8 (range 12‐745) words. The messages addressed topics such as welcoming participants, elaborating on goal setting (module 1), integrating attention training and detached mindfulness into daily routines (modules 2 and 3), and supporting participants in challenging unhelpful metacognitions. No messages were prompted by misunderstandings of the intervention content or technical issues. Four out of 5 test-participants accessed the intervention via smart phone or tablet. No test-participant reported troubles with smart phone access, and no comments related to the description of the values-clarification exercise, of which both were adjusted after the internal field testing. Outcomes are presented in Textbox 1.

Textbox 1. **Outcomes from external field testing.**

**Positive feedback from test-participants**

- “… I am slowly returning to life where cancer doesn’t occupy as much space. Not because it’s forgotten, but more integrated. The tools in your investigation were really useful.” [Male, aged 71 years]
- “I use detached mindfulness regularly. Especially if I’m not occupied with something else at the moment, my mind tends to circle around cancer, and I find that the method helps to stop that.” [Female, aged 43 years]
- “I am incredibly grateful that I was allowed to participate in this program. Even with the bumps along the way, it has been a huge help. In many ways, it’s easier to sit here and write with you than if we were face to face - I like that I have the opportunity to write something, consider, rephrase, etc - or just write freely in a flood of ’unloading,' depending on what I need on that day... Gold!” [Female, aged 51 years]
- “I feel so lucky being part of this program. While I sit here and write, I hear the birds singing. And enjoy it. I live my life fully. I do not postpone things. I know there are late effects, but I will not have them ruin my mood…” [Female, aged 55 years]

**Constructive feedback from test-participants**

- “It could also be good if a notification could be sent in e-boks when there is a new message from my therapist.” [Female, aged 49 years]
- “… There should be some form of notification when there is new activity on the platform.” [Male, aged 71 years]
- “It could be good if one could see how far they are in the program - if the round circles on the front page could possibly change color when a module is completed. I personally know how difficult it is to remember such things when there are several days in between.” [Female, aged 49 years]
- “I have copied my action plan step by step so that I can refer to it if I ‘forget’ it at times. Is it possible to have it compiled for printing at the end of the program? - good to hang up where it is visible in everyday life.” [Female, aged 55 years]
- “…so that one can click into what concerns oneself - sometimes it can be quite depressing to read through a long list of late effects - it's not certain that one has them all oneself, but one is reminded of one's vulnerability. Possibly bubbles on a page under ‘the box’ with ‘fatigue,’ ‘sleep problems,’ etc…” [Female, aged 51 years]

The results were presented to the research team in April 2023. The research team deemed TG-iConquerFear version 2 as satisfactory. The minor suggestions from the external field testing were not implemented due to time and resource limitations but will be considered for future updates.

## Discussion

### Principal Findings

We adapted a self-guided eHealth intervention for FCR into a tailored therapist-guided eHealth intervention aimed at survivors of CRC guided by the Information System research framework. The process was overseen by a multidisciplinary research team including 2 survivors of CRC. Based on knowledge and experience in this team, both minor and major adaptations were made. The addition of an embedded message system facilitating therapist-participant communication was the primary change in the iConquerFear program. However, during field testing it became evident that additional content (eg, the Lighthouse Metaphor or reflective questions) was necessary to optimize adherence and facilitate guidance. Consequently, TG-iConquerFear is more resource demanding than iConquerFear, and we will evaluate the efficacy and cost-effectiveness of these choices, which may limit scalability (ClinicalTrials.gov NCT04287218).

The smooth adaptation of iConquerFear into TG-iConquerFear was greatly facilitated by the extensive research led by the Australian PoCoG evaluating ConquerFear and iConquerFear [26-28,45]. This prior work served as a valuable foundation, allowing us to expedite the launch of the randomized controlled trial, as comprehensive testing of intervention content had already been conducted. Below, we discuss considerations related to the two key requests that emerged from pilot testing of iConquerFear [26] which we addressed in the first round of adaptation to TG-iConquerFear version 1.

### Considerations on How to Enhance “Personal and Relatable” Properties

Some iConquerFear pilot test participants [26] indicated that certain content within the program felt impersonal or unrelatable. Two main directions were considered to promote engagement by tailoring the intervention [46]: targeting a specific gender (men) or targeting a single cancer type (CRC). Choosing a gender-based approach simplifies the selection of colors, images, patients featured in videos, and examples, aiming for enhanced engagement among participants of a specific gender. However, several modules include content pertaining to specific cancer types, for instance, living in alignment with one’s values, where focusing on a specific cancer type such as CRC allows for framing personal examples closely tied to everyday life such as “what if my ostomy leaks?” Similarly, cancer type plays a defining role in shaping the cancer follow-up program, advisory elements, and the identification of alarm symptoms to be monitored. These elements do not apply universally to a specific gender, and one could argue that cancer type is a more defining factor in determining the specific needs reported by cancer survivors, rather than gender alone [47]. For instance, a female survivors of CRC is likely to have far more in common with a male survivor of CRC than a female survivor of breast cancer. By considering the cancer type and adjusting relevant advisory elements, the intervention was customized to meet the unique needs of individuals based on their specific cancer experiences. This tailored approach balances scalability and engagement by ensuring that the content remains relevant, relatable, and meaningful to participants, hopefully enhancing the engagement and effectiveness of the TG-iConquerFear intervention. No end user feedback was received regarding impersonal or unrelatable content after adaptation.

A third option involves the creation of distinct intervention versions with personalized content based on demographic factors such as age, gender, cancer type, or needs, thereby presenting only pertinent content to individual participants although this approach risks relevant content being excluded due to individual differences in patient needs and how they report them. However, the software used for our intervention was incapable of accommodating such customization. Furthermore, implementing this approach would have necessitated a substantial allocation of time and resources beyond our available capacity. Nonetheless, this avenue remains an intriguing prospect for future research endeavors, warranting further exploration and consideration.

### Consideration on How to Add Therapist Guidance

Some participants in the iConquerFear pilot expressed a desire for personal contact with a researcher or clinician [26], which was seen as a potential way to motivate engagement with and potential benefit from the 8‐10 week duration of the program. This is in line with a recent meta-review [46]. The potential benefit of adding guidance was also noted by researchers from the cancer recurrence self-help training trial, who found no effect of CBT-based online self-help training [48]. The choice of asynchronous communication was supported by end users and therapists, and the amount of guidance was consistent through the intervention. Reflective questions were incorporated into TG-iConquerFear version 1 at the therapists’ request after each module to monitor adherence and perceived usefulness. Participants received automated tailored feedback momentarily, in accordance with recommendations from the literature [46,49], allowing them to seamlessly continue with the intervention, and these reflective questions facilitated a dialogue between participant and therapist regarding the usefulness of the module content, and strategies to enhance participant outcomes.

### Other Strategies to Enhance Adherence

#### Easy Access

Difficulties in integrating the intervention into daily life activities, as exemplified by an internal test-participant, have also been reported in the literature as a barrier for adoption and adherence when targeting fatigue [50]. This issue is closely related to the barrier of perceived time burden associated with participating in digital interventions [46]. Given that only minor changes were required to adapt the TG-iConquerFear version 1 to fit a smart-phone layout, this aspect was prioritized.

#### Promoting Competence

In TG-iConquerFear version 1, the value-based model was inadequately explained, leading to a lack of self-competence reported by an internal test-participant. Competence can be promoted by encouraging users to set graded goals with smaller achievable steps, thereby increasing confidence through experiences of success as described in the person-based approach to intervention development [8]. Adjustments were made to clearly outline how to work with goals and values to fully use the module’s potential. Additionally, tailored feedback was provided to congratulate success in goal achievement and offer remedial advice if goals were not attained [8].

#### Reminders

Technology-based reminders (eg, prompts) have the potential to promote engagement with digital interventions [1,46], particularly when participants choose to receive them [51]. Since 2 external test-participants explicitly suggested reminders, this could be considered for future updates. However, it should be noted that no studies in these reviews specifically targeted FCR, and the effect of distributing reminders, which could potentially trigger FCR, in this patient population remains unknown.

The TG-iConquerFear version 2 was deemed ready despite further suggestions for improvement, and in May 2023, a randomized controlled trial investigating TG-iConquerFear versus augmented treatment as usual was launched [52]. The suggestions, though noted, were seen as relatively minor, and addressing them would have required a significant allocation of resources disproportionate to their perceived impact. Given that each cycle typically generates some feedback, the multidisciplinary research team convened to make a decision regarding the intervention’s readiness for the randomized controlled trial.

### Strengths and Limitations

The process of adaptation was guided by an existing framework for information system research. The framework allows for multiple iterative processes, which were needed in this study. It is a strength of this study that the KB comprised insights from multiple sources. The therapists performing the guidance were experienced in delivering anxiety-related online therapy, and the software designers facilitating the adaptation process in the design cycle had expertise in health care software development. The field testing participants were relatively newly diagnosed and the females were younger than the average female patient with colon cancer. Changes due to their comments may have slightly shifted the focus of the final version of the intervention to address a somewhat younger audience. However, as FCR is more prevalent in younger than older survivors [53], and the incidence of CRC in younger patients continues to increase [54], this may be an advantage. Furthermore, we only included 5 participants in the external field testing, but all 5 participants completed all or almost all modules. This study aimed to test the adaptation of an already feasible and pilot tested intervention, which is why the small sample size was accepted. No patients with rectal cancer were included in the test group. We have no reasons to believe that their feedback would be any different as needs and late effects are comparable [55,56].

### Clinical Implications

This study demonstrates a process to adapt a self-guided eHealth intervention into a tailored therapist-guided eHealth intervention, which could help efficiently address survivorship concerns such as FCR. Guided eHealth interventions are effective supplements to face-to-face intervention and could be a valuable step in a stepped-care model, where self-guided interventions might be the first step for survivors with mild symptoms [57,58]. Guided interventions require some level of involvement of health personnel, but can significantly increase the accessibility and reach of psychological interventions while promoting engagement and efficacy [14]. Determining the optimal dose of guidance for the severity of symptoms is needed [29].

### Future Directions

The quality and content of therapist guidance will be assessed, alongside the investigation of TG-iConquerFear’s efficacy in a larger, more diverse population across Denmark [52]. Additionally, further evaluation of TG-iConquerFear’s performance across various devices, including smartphones or iPhone, tablets or iPads, and computers, will help refine its usability and ensure compatibility across platforms, thereby increasing scalability. Future studies should investigate the usability of eHealth interventions such as TG-iConquerFear for patients experiencing late effects such as fatigue and peripheral neuropathy, particularly in relation to content load and the touchscreen-based exercises. Finally, linking TG-iConquerFear with health apps or smartwatches could provide valuable insights into the usage of the intervention combined with, for example, participant activity levels and heart rate, allowing for an evaluation of the interplay between intervention content and bio-physiological and behavioral parameters.

## Conclusion

It is possible to successfully adapt a self-guided eHealth intervention for people with FCR into a tailored therapist-guided intervention. This paper provides an overview of the process and lists considerations based on experience. The described procedure can be used in similar settings where the wish is to incorporate guidance in an existing self-guided eHealth intervention.

## Supplementary material

10.2196/63486 Multimedia Appendix 1 All primary changes.

10.2196/63486 Multimedia Appendix 2 The lighthouse metaphor and exercise.

## ABBREVIATIONS

CRC: colorectal cancer
FCR: fear of cancer recurrence
KB: knowledge base
PoCoG: Psycho-Oncology Co-Operative Research Group
TG-iConquerFear: Therapist Guided iConquerFear
