# Supplementary material for: Adapting a Self-Guided eHealth Intervention Into a Tailored Therapist-Guided eHealth Intervention for Survivors of Colorectal Cancer
Source: JMIR Cancer. 2025 Mar 5;11:e63486. doi: 10.2196/63486 (PMC11900901; doi:10.2196/63486)
Supplement: Multimedia Appendix 1 [file cancer-v11-e63486-s001.docx]

# Generelle ændringer / general changes

| **Type af ændring** | **Dansk beskrivelse af ændringen**  Danish description of the change | **English description of the change**  English description of the change |
| --- | --- | --- |
| **Fjernelse af tekst/ information/ funktioner**  Removal of tekst/ information/ functions | Det er ikke angivet i toppen af hver modulside, hvilket modul man er i. | It doesn’t say which module you’re in on the top of each page. |
|  | Det er ikke angivet hvilket modul, pjecerne hører til. | It doesn’t say what module the handouts are connected to on the top of handouts. |
|  | Programmet vil kun kunne bruges på computer (og ikke tablet eller mobil), derfor er beskrivelser ift. dette slettet i teksten. | The program will only function on computer (and not tablet or mobile phone) therefor descriptions regarding this have been discarded. |
| **Andre indholdsændringer**  Other content changes | Navnet på programmet er ændret. Den danske version af behandlingsprogrammet hedder ’LivEFTERkræft’ | The name of the program has been changed to ‘LivEFTERkræft’. The name can be translated to “LifeAFTERcancer”. |
|  | Alt, der har været skrevet med versaler, er ændret til at være skrevet med minuskler (små bogstaver). | Everything written in capital letters is changed to lower-case letters. |
|  | Alle videoer udskiftes til videoer med danske læger og kræftoverlever. | All videos are replaced with videos of Danish doctors and cancer survivors. |
|  | Alle udråbstegn er slettet. | All exclamation marks have been deleted. |
|  | Personnavne i eksempler er ændret til mere almene danske navne | Names in examples have been changed to more common Danish names. |
|  | Ved udfyldelse af øvelser og besvarelse af spørgeskemaer og sliding scales vil svaret være tilgængeligt for psykologen. Knappen vil derfor i nogle tilfælde hedde ’send’ i stedet for ’gem’.  Den foregående tekst vil i disse tilfælde hedde ”har du sendt dit svar?” i stedet for ”har du gemt dit svar?”. | The psychologist can access the participants’ answers in exercises, questionnaires and. Sliding scales. Therefore, that button ‘save’ will sometimes be named ‘send’ instead.  The preceding text will in this instance read “have you send your answer?” instead of “have you saved your answer?”. |
|  | De billeder, der findes gennem programmet, er valgt ud fra at skulle være så ens med billederne fra iConquerFear som muligt, men lidt mere naturprægede og hovedsageligt med personer, der aldersmæssigt tilsvarer EFTERs primære målgruppe. | The pictures in the program are sought to be as similar as possible to the pictures in iConquerFear but with more nature and mostly featuring people from EFTERs primarily targeted age group. |
|  | Den tilknyttede psykolog har adgang til deltagerens mindfulness-dagbog. | The participants psychologist has access to the participants mindfulness-diary. |
|  | Funktionen ’Redskaber-på-farten’ er omdøbt til bare at hedde ’Redskaber’. Funktionen placeres i skærmens top, hvorfor beskrivelsen af placeringen ændres. | The function name of ’Tools-on-the-go’ is changed to ‘Tools’. The function is placed in the top of the screen in this program and therefore the description of the placement of the function is changed. |
|  | Noten, der informerede om, at burgeren blev logget ud efter en times inaktivitet, er slettet i alle moduler. I stedet er det skrevet ind i velkomstmodulet side 2. | The note that informed the users that they would be logged out after an hour of inactivity has been deleted in all modules. The information will appear on page 2 in the welcome module instead. |

# Velkomstmodul / welcome module

| **Type af ændring** | **Dansk beskrivelse af ændringen**  Danish description of the change | **English description of the change**  English description of the change |
| --- | --- | --- |
| **Tilføjelse af tekst/information**  Addition of tekst/information  Side 1 / Page 1 | *Du vil have den samme psykolog tilknyttet gennem hele forløbet. Din psykolog er til for at hjælpe dig og guide dig på rette vej. Psykologen kan motivere dig og fastholde dig, når du møder udfordringer. For at optimere samarbejdet har psykologen adgang til dine øvelser og de svar, du angiver i programmet, når du trykker "send". Psykologen har tavshedspligt.  Du kan altid sende beskeder til din psykolog via "besked"-funktionen, ligesom denne kan sende beskeder til dig. Du kan forvente at høre fra din psykolog minimum på ugentlig basis.* | *You will be affiliated to the same psychologist throughout the program. Your psychologist will help you and set you right. The psychologist can motivate you and help you stick to the program when you meet obstacles. In order to optimize the co-operation, the psychologist has access to your excises and answers thought the program, when you click “send”. The psychologist is pledged to secrecy.*  *You can always send messages to your psychologist via the “message”-function and your psychologist can send messages to you. You can expect to hear from your psychologist at least once a week.* |
| **Fjernelse af tekst/information**  Removal of tekst/information  Side 1 / page 1 | *Disse strategier er vist at være effektive, når de gives som en del af en ansigt-til-ansigt-terapi.* | *These strategies have been shown to be effective when delivered as part of a face-to-face therapy.* |
| **Andre indholdsændringer**  Other content changes  Side 1 / page 1 | *LivEFTERkræft hjælper dig til at lære* ***strategier*** *til håndtering af din frygt for tilbagefald af kræft.*  Er ændret til:  *LivEFTERkræft hjælper dig til at lære* ***konkrete redskaber*** *til håndtering af din frygt for tilbagefald af kræft.* | *iConquerFear will help you learn* ***strategies*** *for managing your fear of cancer recurrence.*  Has been changed to:  *iConquerFear will help you learn* ***concrete tools*** *for managing your fear of cancer recurrence.* |
| Side 1 / page 1 | *Vi anbefaler, at du arbejder dig igennem LivEFTERkræft i løbet af 10****-12*** *uger.*  Er ændret til:  *Vi anbefaler, at du arbejder dig igennem LivEFTERkræft i løbet af 10 uger.* | *We recommend you work through iConquerFear over 10 to 12 weeks.*  Has been changed to:  *We recommend you work through iConquerFear over 10 weeks.* |
| Side 2 / page 2 | Teksten vedr. hvordan programmet virker er blevet frokortet og tilpasset den danske platforms format. | The text regarding how the program works has been shortened and adjusted to the format of the danish platform. |
| Side 4/ Page 4 | *At overveje fordele og ulemper BÅDE ved at ændre dig og ved at forblive den samme kan hjælpe dig med at beslutte, om du er klar til* ***at forpligte dig til iConquerFear***.  Er blevet ændret til:  *At overveje fordele og ulemper, både ved at ændre dig og ved at forblive den samme, kan hjælpe dig med at beslutte, om du er klar til* ***forandring****.* | *Thinking through the pros and cons of BOTH changing and staying the same can help you decide whether you’re* ***ready to commit to iConquerFear****.*  Changed to: *Thinking through the pros and cons of both changing and staying the same can help you decide whether you’re* ***ready to change****.* |
| Side 5 / page 5 | *Jo mere du engagerer dig i LivEFTERkræft -programmet, jo mere får du ud af det.*  *For at få mest muligt ud af iConquerFear:*   - *gennemgå hvert modul* - *udfør aktiviteterne i modulerne* - *udfyld spørgeskemaerne*   Er ændret til:  *Jo mere du engagerer dig i LivEFTERkræft -programmet, jo mere får du ud af det.*  *Hvis du er klar til at håndtere dine bekymringer for tilbagefald af kræft, kan du klikke nedenfor.* | *The more you engage with the iConquerFear program the more you will get out of it. To get the most out of iConquerFear please:*   - *look through each module* - *practice the module activities* - *complete the assessment questionnaires*   Has been changed to:  *The more you engage with the iConquerFear program the more you will get out of it.*  *If you are ready to handle your worries of cancer coming back, you can click below.* |

# Modul 1 / module 1

| **Type af ændring** | **Dansk beskrivelse af ændringen**  Danish description of the change | **English description of the change**  English description of the change |
| --- | --- | --- |
| **Tilføjelse af tekst/information**  Addition of tekst/information  Side 6  Afsnit: Hvilke tanker og følelser synes at forhindre dig i dette mål  /  Page 6  Section: What thoughts and feelings seem to stop you from achieving that goal? | *Hvad hvis jeg får ondt i maven? Hvad hvis min stomi lækker?, Hvad hvis vi møder nogen, vi kender?, Hvad hvis vi ikke har noget at tale om?* | *What if I get stomach pains? What if my ostomy leaks? What if we meet someone we know? What if we don’t have anything to talk about?* |
| Side 6  Afsnit: Vælg nu en eller flere handlinger, der vil hjælpe dig til at nå dette mål. /  Page 6:  Section: Now pick an action(s) that will lead you to accomplish that goal. | *Et eksempel på en handling er "book bord til næste lørdag på din yndlingsrestaurant" eller ”****Pak en taske til nødssituationer” eller ”Vælg en restaurant i en anden by”.*** | *Now pick an action(s) that will lead you to accomplish that goal. An example of an action is to "book dinner at our favourite restaurant for this saturday night"* ***or “pack a bag to emergencies” or “pick a restaurant in another city.”.*** |
| **Andre indholdsændringer**  Other content changes  Mål / Goals: | *I dette modul lærer du:*  Er ændret til:  *I dette modul skal vi se på hvilke værdier, der er vigtige for dig i dit liv EFTER kræft* | *In this module you will learn:* Has been changed to:  *In this module we will take a look at the values important to you in your life AFTER cancer.* |
| Side 4/ Page 4: | ***Angsten for igen at skulle se kræften i øjnene giver en mulighed for igen at*** *få kontakt med de ting, der er virkelig vigtige;*  Er ændret til:  ***At se kræften i øjnene, giver mulighed for at genetablere*** *kontakten med de ting, der er virkelig vigtige;* | ***The turmoil of facing*** *cancer provides an* *opportunity to re-connect with those things that are really important;*  Has been changed to:  ***Facing cancer provides an opportunity to re-connect*** *with the things that are really important;* |
| Side 6  OBS. tilsvarende ændring er lavet i modul 5 side 1. / Page 6  N.B. a similar changed is made in module 5 page 1. | *Negative* ***udsagn*** *(fx "Jeg bliver aldrig god til dette", "****Hvad er formålet med at forsøge****?") baserer sig ofte på,* ***at du kan forudsige fremtiden, og at du ignorerer dine tidligere succeser.***  Er ændret til:  *Negative* ***tanker*** *(fx "****Alle kan se jeg har en stomi",*** *"Jeg er ikke god til at samtale” eller ”****Vi går altid tidligt hjem, fordi jeg bliver træt****”) baserer sig ofte på,* ***at du fokuserer på tidligere fiaskoer i stedet for tidligere succeser.*** | *Negative evaluations (e.g. “I’ll never be any good at this”, “****What is the point in trying?****”) often* ***assume you can predict the future and ignore your past successes****.*  Has been change to:  *Negative evaluations (e.g. “****everyone can see I have a ostomy****”, “I’ll never be any good at this”, or “****we always go home early because I get tired****”) often* ***stems from focusing on past fiascoes instead of past successes.*** |

# Modul 2 / module 2

| **Type af ændring** | **Dansk beskrivelse af ændringen**  Danish description of the change | **English description of the change**  English description of the change |
| --- | --- | --- |
| **Tilføjelse af tekst/information**  Addition og tekst/information  Side 4 / Page 4 | De spørgeskemaer, der fremgår i modul 4 er tilføjet til også at indgå i modul 2. Bemærk det kun er 1 af spørgeskemaerne fra iConquerFear og at der er tilføjet en sliding scale. Det vil fremgå som følger:  *Disse spørgsmål vedrører tanker og forestillinger, som man kan gøre sig angående ens egen måde at tænke på. Nedenfor er der en række overbevisninger, folk tidligere har udtrykt. Læs hvert enkelt udsagn og noter, hvor er enig/uenig du er. Besvar alle spørgsmålene – der er ingen rigtige eller forkerte svar.*   - *Min bekymringstendens fortsætter, uanset hvordan jeg prøver at stoppe den* - *Hvis jeg først begynder at bekymre mig, kan jeg ikke stoppe igen* - *Jeg kunne gøre mig selv syg ved at bekymring mig* - *Jeg kan ikke ignorere min tendens til at bekymre mig* - *Min bekymringstendens kunne gøre mig vanvittig* - *Det er farligt for mig, at jeg bekymrer mig*   *Sliding scale:*  *På en skala fra 1-100, hvor stor mener du selv risikoen er for at du får et tilbagefald af din kræftsygdom?* | The questionnaires in module 4 will also figue in module 2. Note that only 1 of the questionnaires from IConquerFear are included and there is added a sliding scale. It will appear as follows:  *This survey is concerned with beliefs people have about their thinking.*  *Below, there’s a string of beliefs other people have expressed. Read each statement and note how much you agree. Answer all the questions - there are no right or wrong answers.*   \| - *My worrying is dangerous for me* \|  \|  \|  \|  \| \| --- \| --- \| --- \| --- \| --- \| \| - *I could make myself sick with worrying* \|  \|  \|  \|  \| \| - *My worrying thoughts persist, no matter how I try to stop them* \|  \|  \|  \|  \| \| - *I cannot ignore my worrying thoughts* \|  \|  \|  \|  \| \| - *My worrying could make me go mad* \|  \|  \|  \|  \| \| - *When I start worrying, I cannot stop* \|  \|  \|  \|  \|   *Sliding scale:*  *How high do you think the risk of cancer recurrence is on a scale between 1 and 100?* |

# Modul 3 / module 3

| **Type af ændring** | **Dansk beskrivelse af ændringen**  Danish description of the change | **English description of the change**  English description of the change |
| --- | --- | --- |
| **Tilføjelse af tekst/information**  Addition of tekst/information  Pjece: Frakoblet opmærksomhed.  Afsnit: ’Verbal gentagelse’ /  Participant handout: Dethatched mindfulness.  Section: ‘Verbal Loop’. | *Eksempelvis kan du gentage tanken ”Jeg kommer til at opleve et tilbagefald af kræft” for dig selv. Når du gentager den for dig selv, skal du kun forholde dig til tankens lyde og dermed ikke forholde dig til dens indhold og betydning, og samtidig huske dig selv på, at tanker bare er tanker, og ikke sandheder.* | *You could for example repeat the thought “I will experience a recurrence of cancer” to yourself. As you repeat it to yourself you should only engage with the sounds of the thought, and not engage with the content or meaning of the thought, and at the same time remind yourself that thoughts are just thoughts and not truths.* |
| **Fjernelse af tekst/information**  Removal of tekst/information  Side 1/ page 1 | *Godt forsøg. Selvom det måske ser ud til på kort sigt at virke at adsprede dig selv fra dine bekymringer om tilbagefald af kræft* ***ved at kæmpe med at skubbe bekymringerne væk****, fastholder du faktisk følelsen af den trussel, som dine bekymringer udgør, hvilket øger din bekymring på lang sigt.*  Er ændret til:  *Selvom det måske ser ud til på kort sigt at virke at adsprede dig selv fra dine bekymringer om tilbagefald af kræft eller undgå disse tanker, fastholder du faktisk følelsen af den trussel, som dine bekymringer udgør, hvilket øger din bekymring på lang sigt.* | *Nice try. While avoiding or distracting yourself from worries about your cancer coming back may seem to help in the short-term,* ***by struggling to push the worries away,*** *you’re actually maintaining the sense of threat posed by those worries, causing you greater distress in the long run.*  Is changed to:  *While distracting or avoiding yourself from your worries of cancer coming back it might seem to help in the short term, you’re actually maintaining the sense of threat posed by those worries, causing you greater distress in the long run.* |
| Side 3 / page 3 | *Det kan være, du synes, at det at føre frakoblet opmærksomhed -dagbog hjælper dig til at reflektere over, hvor godt frakoblet opmærksomhed fungerer for dig.*  Is changed to:  *Det kan være, du synes, at det at føre dagbog hjælper dig til at reflektere over, hvor godt frakoblet opmærksomhed fungerer for dig. For at kunne følge med i din proces og hjælpe dig på bedste vis, har din psykolog adgang til din dagbog.* | *You may find completing this detached mindfulness diary helps you reflect on how well detached mindfulness is working for you.*  Is changed to:    *You may find completing this diary helps you reflect on how well detached mindfulness is working for you. You psychologist has access to your mindfulness dairy in order to keep track of your process and help you the best.* |

# Modul 4 / module 4

| **Type af ændring** | **Dansk beskrivelse af ændringen**  Danish description of the change | **English description of the change**  English description of the change |
| --- | --- | --- |
| **Tilføjelse af tekst/information**  Addition of tekst/information  Side 5 (tidligere side 6) / page 5 (former page 6) | Der er tilføjet en sliding scale mellem 1 og 100 efter de to spørgeskemaer med teksten: På en skala fra 1-100, hvor stor mener du selv risikoen er for at du får et tilbagefald af din kræftsygdom? | Following the two questionnaires there is added a sliding scale between 1 and 100 with the text:  How high do you think the risk of cancer recurrence is on a scale between 1 and 100? |
| Pjece: Sund livsstil  Afsnit: sund kost /  Participant handout: healthy eating/lifestyle  Section: healthy eating | *Der er en bred enighed om, at kost har en betydning for udviklingen af tarmkræft*. …. *Indtag af grøntsager er den faktor, som i flest studier har vist sig at være forbundet med nedsat risiko for tarmkræft. Fundene vedrørende indtagelse af frugt er mindre klart sammenhængende med nedsat risiko. Derudover har en metanalyse (dvs. en sammenlignende analyse af allerede eksisterende studier) af 13 case-kontrolstudier vist mangel på sammenhæng mellem totalt fedtindtag og tarmkræft. Det vil sige, der ikke er evidens for en sammenhæng mellem fedtindtag og forøget risiko for tarmkræft.*  *Sammenhængen mellem kødindtagelse og risiko for kræft, er undersøgt adskillige gange. Flere studier (heriblandt, ”Nurse Health Study” og ”Male Health Professionals Study”) har vist sammenhæng mellem hyppig kødindtagelse og tarmkræft. Andre studier har ikke vist denne sammenhæng. En metaanalyse af 24 studier har dog vist en relativ forøget risiko for tarmkræft for personer med højest versus lavest indtag af rødt eller forarbejdet kød.* | *There is a broad agreement that diet is important in relation to developing colorectal cancer…. The factor that most studies have found to be connected to recued risk of colorectal cancer is vegetable consumption. The results regarding fruit consumption is not as clearly associated with reduced risk. A meta-analysis (an analysis comparing already existing studies) of 13 case-controlled studies has also shown lack of association between total fat consumption and colorectal cancer. That means that there is no evidence of an association between total fat consumption and increased risk of colorectal cancer.*  *The relation between meat consumption and risk of cancer har been examined several times. Multiple studies (including “Nurse Health Study” and “Male Health Professionals Study”) has shown a relation between frequent meat consumption and colorectal cancer. Other studies have not found this connection. A meta-analysis of 24 studies has shown a relative increased risk of colorectal cancer for people with high versus low consumption of red or processed meat.* |
| Pjece: Sund livsstil  Afsnit: kostanbefalinger, slutteligt /  Participant handout: healthy eating/lifestyle  Section: dietary recommendations, as an ending remark | *Hvis du har nedsat appetit eller har tabt dig, kan det også være en god ide at tale med din læge eller en sygeplejerske om, hvad du kan gøre.* | *If you have reduced appetite or have lost weight it can also be a good idea to talk to your doctor or a nurse about what you can do.* |
| Pjece: Sund livsstil  Afsnit: kostanbefalinger /  Participant handout: healthy eating/lifestyle  Section: dietary recommendations | **Tabel S1.** Forebyggelsesmuligheder for tarmkræft.   \|  \| ***Evidens-niveau*** \| \| --- \| --- \| \| *Øget indtag af grøntsager og frugt* \| *IIa* \| \| *Nedsat indtag af animalsk fedt og kalorier* \| *IIa* \| \| *Øget fysisk aktivitet* \| *IIa* \| \| *Ryge- og alkoholophør* \| *IIa* \| \| *NSAID/acetylsalicylsyre-brug* \| *Ib** \| \| *Calcium-indtag* \| *IIa/Ib*** \|   *Styrken af evidens angives i niveau fra 1 til 4, hvor 1 er højeste niveau. *anbefales pt. ikke da der er usikkerhed om, at risikoen for alvorlige bivirkninger opvejer den kræftforebyggende* *effekt.*  ***gælder adenomer*  *Kilde: DCCG’s nationale retningslinier for diagnostik og behandling af kolorektal cancer:primær forebyggelse ad kolorektal cancer, DCCG, 1. 12. 2016.* | **Table S1.** Prevention of colorectal cancer.   \|  \| ***Evidence-level*** \| \| --- \| --- \| \| *Increased intake of fruits and vegetables* \| *IIa* \| \| *Reduced intake of animal fats and calories* \| *IIa* \| \| *Increased physical activity* \| *IIa* \| \| *Termination of smoking and drinking alcohol* \| *IIa* \| \| *NSAID/acetylsalicylsyre use* \| *Ib** \| \| *Calcium intake* \| *IIa/Ib*** \|   *The streght of the evidence is indicated in levels from 1 to 4 where 1 is the highest level.*  **At preset this isn’t recommended due to uncertainty regarding whether the risk of serious side effects excess the preventing effect.*  ***doesn’t apply to adenoma*  *Source: DCCG’s nationale retningslinier for diagnostik og behandling af kolorektal cancer:primær forebyggelse ad kolorektal cancer, DCCG, 1. 12. 2016.* |
| Pjece: Sund livsstil  Afsnit: nyt afsnit efter kostanbefalinger  /  Participant handout: healthy eating/lifestyle  Section: new section after dietary recommendations | ***3. Kost og stomi***  *Har du stomi, er der nogle tommelfingerregler du kan følge, for at forbedre din fordøjelse. Du kan læse Kræftens Bekæmpelses generelle kostanbefalinger ved stomi og anbefalinger specifikt for kolostomi og ileostomi nedenfor.*  *Hvis du oplever diarré og luft i maven kan du prøve at spise lidt mindre (men ikke undgå):*   - *Brød med mange kerner og fuldkornsprodukter f.eks. rugbrød* - *Fed mad, særligt smør, fløde, cremefraiche, olie og mayonnaise* - *Grøntsager og frugt* - *Kaffe og alkohol* - *Tyggegummi og pastiller med kunstigt sødemiddel (sorbitol)* - *Nogle får diarré ved indtagelse af mælk og mælkeprodukter, fordi kræftbehandlingen har givet midlertidig laktoseintolerance. Her kan man vælge laktosefri produkter.* - *Hvis du har meget luft i maven kan det forværres af blomkål, porrer, løg, ærter, hvidløg, jordskokker og tørrede bælgfrugter*   *Specifikke kostanbefalinger ved kolostomi;*  *Du vil normalt kunne spise en almindelig, sund kost, som bør være fedtfattigt og fiberholdigt med groft brød, frugt og grønt fordelt på 3 hoved- og 2 mellemmåltider i døgnet.*  *Specifikke kostanbefalinger ved midlertidig ileostomi:*  *Du kan som hovedregel spise normalt. Du kan dog opleve væsketab. Her er det en god ide at drikke rigeligt med væske, tilskud af buillon og at salte din mad lidt rigeligt.*  *Specifikke kostanbefalinger ved permanent ileostomi;*  *Hvis der er normalt output til stomiposen, anbefales det at:*   - *Spise langsomt og tygge maden grundigt* - *Indtage rigelig væske - mindst 2-3 liter dagligt* - *Spise flere små, regelmæssige måltider i løbet af dagen* - *Undgår eller nedsæt mængden af trevlede grøntsager (f.eks. porre og asparges), champignon, tørrede ærter og bønner, pølseskind og hinder fra citrusfrugter* - *Du kan eventuelt forsøge med et tilskud af HUSK® Psylliumfrøskaller, som fås på apoteket, hos Matas og i en del supermarkeder*   *Hvis der er problemer med stort output til stomiposen, anbefales det at:*   - *Nedsæt mængden af fiberrige fødevarer såsom rugbrød, grøntsager og frugt* - *Indtage andre væsker end vand* - *Undgå at indtage væske til måltiderne* - *Drikke væske, der indeholder salt, f.eks. mælk, bouillon og Revolyt* - *Drikke mindre kaffe og te samt juice, saft og sodavand, som ikke indeholder salt*   *Der er enkelte fødevarer, der kan danne prop i stomien: Det er især fødevarer, der er trevlede såsom asparges, har sejt skind som fx. pølseskind eller frugt med faste hinder som fx en appelsin.*  *Sørger du for at tygge maden grundigt og skære den i små stykker, er risikoen for prop i stomien meget lille.*  ***4. kost og dumping syndrom***  *Kræftens Bekæmpelse har udarbejdet kostråd til en antidumping-diæt:*   - *Spis 6-8 små måltider i løbet af dagen* - *Spis langsomt og tyg din mad grundigt* - *Sid op, når du spiser. Hvis du har behov for det, kan du ligge ned efter måltidet.* - *Begræns eller udelad sukker og sukkerholdige fødevarer - særligt i flydende form som for eksempel sodavand, saftevand, juice, milkshake, slik, chokolade, is, kager, honning, marmelade, frugtgrød og frugtsupper* - *Det er individuelt, om man kan tåle at drikke og spise mælk og mælkeprodukter. Hvis du spiser mælkeprodukter, så vælg produkter uden tilsat sukker.* - *Undgå at drikke til måltiderne, men drik gerne en halv time før og efter måltidet*   *Spis gerne:*   - *Fuldkorn, pasta, kartofler, ris, grøntsager, frugt og bær (kulhydrater)* - *Kød, fisk, fjerkræ, æg og bønner/linser (protein)* - *Smør, olie, fede oste, mayonnaise og alle slags nødder og kerner (fedt). Det kan være en god ide at spise fedtholdigt, hvis du oplever dumping, fordi fedtstofferne kan medvirke til at mavesækken tømmes langsommere.*   ***5. Fedme***  *Der er sikkert bevis for at der findes sammenhæng mellem fedme (BMI >30, og særligt fedt på maven), metabolisk syndrom og risiko for tarmkræft. Denne sammenhæng gælder særligt for mænd.*  *Det vides også at der er en sammenhængen mellem fysisk aktivitet og nedsat risiko for tarmkræft. Selv moderat fysisk aktivitet har vist nedsat risiko. Fysisk aktivitet påvirker også hormonproduktionen og immunsystemet gavnligt.* | ***Diet and ostomy***  *If you have an ostomy, there are some guidelines you can follow to improve your digestion. Below, you can read the general dietary recommendations for ostomy and specifically for colostomy and ileostomy devised by Kræftens Bekæmpelse.*  *If* *you experience diarrhea and intestinal gas you can try eating less (but not avoid):*   - *Bread with many seeds and whole grain products for example rye bread* - *Foods with a high density of fat for example butter, cream, creme fraiche, oil and mayonnaise* - *Vegetables and fruit* - *Coffee and alcohol* - *Chewing gum and mints with artificial sweeteners (sorbitol)* - *A few get diarrhea from consumption of milk and milk products because the cancer treatment has created a temporary lactose intolerance. If so, you can choose lactose free products.* - *If you experience a lot of intestinal gas it can be worsened by cauliflower, leeks, onions, peas, Jerusalem artichokes and dried legumes*   *Specific dietary recommendations for colostomy:*  *Normally you will be able to eat an ordinary healthy diet preferably low on fat and high on fiber with coarse bead, fruits and greens, divided between 3 main meals and 2 snacks a day.*  *Specific dietary recommendations for temporary ileostomy:*  *Most often you can eat as normal. But you can experience fluid loss. If so, it is a good idea to drink plenty of water, supplemented with bullion and to add extra salt to your food.*  *Specific dietary recommendations for permanent ileostomy:*  *If you have a normal output to the ostomy bag it is recommended to:*   - *Eat slowly and chew the food thoroughly* - *Consume plenty of liquids– as a minimum 2-3 liters daily* - *Eat many small, regular meals during the day* - *Avoid or reduce the amount of fibrous vegetables (i.e. leeks and asparagus), champignon, dried peas and beans, sausage casings and membranes from citrus fruits* - *Putatively you can try taking a supplement of HUSK® Psyllium shells that you can get at the pharmacy, at Matas and in many grocery stores*   *If you struggle with large outputs to the ostomy bag it is recommended to:*   - *Reduce the amount of high-fiber foods such as rye bread, vegetables and fruit* - *Consume other liquids than water* - *Avoid drinking during meals* - *Drink liquids containing salt i.e. milk, bouillon and Revolyt* - *Drink less coffee, tea, juice and soda that do not contain salt*   *There are certain foods that can generate a plug in the ostomy: That is especially fibrous foods such as asparagus, foods that have a tenacious membrane i.e. sausage casing or fruits with membranes such as oranges.*  *If you make sure to chew your food thoroughly and cut it into small pieces the risk of getting a plug in the ostomy will be very low.*  ***4. diet and dumping syndrom***  *Kræftens Bekæmpelse have proposed some dietary recommendations for an anti-dumping diet:*   - *Eat 6-8 small meals during the day* - *Eat slowly and chew your food thoroughly* - *Sit upright when you eat. If needed you can lay down after the meal.* - *Limit or avoid sugar and sugary foods – especially liquids such as sodas, juice, milkshakes, chocolate, ice, cakes, honey, marmalade, stewed fruits, candy and fruit soups.* - *It is individual whether you tolerate dairy products. If you eat or drink dairy, then choose products without added sugars.* - *Avoid drinking during meals but drink half an hour before and after the meal.*   *Do eat:*   - *Whole grain, pasta, potatoes, rice, vegetables, fruit and berries (carbs)* - *Meat, fish, poultry, eggs and beans/lentils (protein)* - *Butter, oil, high fat cheeses, mayonnaise, and all kinds of nuts and seeds (fat). If you experience dumping it a can be a good idea to have a high fat diet because it can contribute to slow down the emptying of the stomach.*   ***5. Obesity***  *There is evidence of a relation between obesity (BMI > 30 and in pedicular abdominal fat), metabolic syndrome and risk of colorectal cancer. This connection mostly applies to men. It is known that there is a relation between physical activity and reduced risk of colorectal cancer. Even physical activity at a moderate level is shown reduced risk. Physical activity also has a positive influence on the hormone production and the immune system.* |
| Pjece: Overlevelsesstatistikker – hvad betyder de?  Afsnit: hvor præcise er statistikkerne /  Participant handout: Making sense of survival statistics  Section: how accurate are the statistics | *Netop dette er grundlaget for tarmkræftscreeningsprogrammet, hvor formålet er at opspore kræften i et tidligt stadie, hvor sygdommen er lettere at behandle* | *This is exactly the rational for the national screening program, where the object is to detect the cancer at an early stage where the disease is easier to treat* |
| Pjece: Overlevelsesstatistikker – hvad betyder de? Afsnit: bliver overlevelsesraterne bedre og bedre /  Participant handout: Making sense of survival statistics  Section: are the survival rates improving? | *Det tager mange år at indsamle data til statistikker, og derfor er statistikkerne som regel lidt forældede. Fx, for en person, der er diagnosticeres med kræft i* ***2016****, kan den angivne 5-årsoverlevelsesrate være baseret på tal fra* ***2012. Statistikken vedrørende tarmkræft er også påvirket af at man i 2014 begyndte at tilbyde screening for tarmkræft for alle personer i alderen 50-74 år.*** *Eftersom kræftbehandling hele tiden forbedres, er udsigterne sandsynligvis bedre, end de var i* ***2012****. Det kan være beroligende at vide, at statistikker, der angiver overlevelsesrater på 5 år, sandsynligvis vil ændre sig og blive forbedret for mange kræftformer de næste 20 år* ***pga. forbedret diagnostik, behandling og opfølgning.*** | *Statistics take many years to collect and therefore are usually slightly out of date. For example, for a person diagnosed with cancer in* ***2016****, the five-year survival rate available may be from* ***2012****.* ***The statistics regarding colorectal cancer is also influenced by the national screening program offered to everyone in the age 50-74 started in 2014.*** *With cancer treatments improving all the time, the outcome is likely to be better than it would have been reported in* ***2012****. It may be reassuring to know that statistics such as five-year survival rates are likely to change and improve for many types of cancers in the next 20 years* ***because of improved diagnostics, treatment and follow up care.*** |
| Pjece: Overlevelsesstatistikker – hvad betyder de?  Afsnit: bliver overlevelsesraterne bedre og bedre /  Participant handout: Making sense of survival statistics  Section: are the survival rates improving? | Generelt **for tarmkræft** gælder det, at jo længere tid der går, jo mindre sandsynligt er det, at kræften kommer tilbage. | Regarding **colorectal cancer** in general the more time that goes by, the less likely it is that the cancer will come back. |
| Pjece: Senfølger /  Participant handout: long-term side effects | Der er tilføjet en yderligere pjece til deltagerne vedrørende senfølger. Det findes i et separat word-dokument kaldet ’translated handouts’. | There is added an extra handout to the participants regarding the long-term side effects. It is found in a separate word document called ‘Translated handouts’. |
| Pjece: Screening /  Participant handout: screening | Der er tilføjet en yderligere pjece til deltagerne vedrørende screening. Det findes i et separat word-dokument kaldet ’translated handouts’. | There is added an extra handout to the participants regarding the Danish screening program. It is found in a separate word document called ’translated handouts’. |
| **Fjernelse af tekst/information**  Removal of tekst/information  Side 3 / page 3 | Al tekst/information, der vedrører andre kræfttyper end tarmkræft er blevet slettet. | All text/information regarding all other types of cancer than colorectal cancer has been deleted |
| Pjece: Yderligere information /  Participant handout: | Pjecen ”yderligere information” er slettet. | The handout ”soruces of further information” har been deleted. |
| Pjece: opfølgende behandling /  Participant handout: follow up care | *I tiden efter at behandlingen er afsluttet, kan man nemt glemme, hvad der er blevet talt om vedr. opfølgning, da det kan virke mindre vigtigt end at modtage aktiv behandling.* | *In the busy time after treatment is finished, it is easy to forget what has been discussed about follow-up as it may seem less critical than receiving active treatment* |
| Pjece: opfølgende behandling/  Participant handout: follow up care | *Fx ved brystkræft har forskning vist, at regelmæssige scanninger, røntgenstråler og blodprøver ikke øger langtidsoverlevelsen, så dette er ikke anbefalet som en del af den opfølgende standardbehandling.* | *In the case of breast cancer, for example, research has shown that having regular scans, X-rays and blood tests does not improve long-term survival, so these are not recommended as part of routine follow-up care.* |
| Pjece: opfølgende behandling /  Participant handout: follow up care | *(fx at spørge til ny kræftbehandling man har hørt om i medierne)* | *(for example to ask about reports in the media about a new cancer treatment)* |
| Pjece: solbeskyttelse/  Participant handout: sun protection | Al tekst/information er slettet. | All text/information has been deleted |
| Pjece: Sund livsstil /  Participant handout: healty eating (healthy lifestyle) | *Et beskedent vægttab for personer, der er overvægtige efter en kræftbehandling, kan også nedsætte risikoen for tilbagevendende kræft* | *Modest weight loss, for those who are overweight following a diagnosis of cancer, may also reduce the risk of cancer recurrence.* |
| Pjece: Sund livsstil  Afsnit: alkohol /  Participant handout: healty eating (healthy lifestyle)  Section: alcohol | ”450 ml. letøl” i passagen:  *En genstand er 10 g alkohol og er lig med 285 ml øl med almindelig alkoholprocent,* ***450 ml letøl****, 100 ml vin eller 30 ml spiritus.* | “450 mL of low alcohol (light) beer” in the passage:  *A standard drink contains 10g alcohol, and is equal to 285mL full strength beer,* ***450mL of low alcohol (light) beer****, 100mL wine and 30mL spirits* |
| Pjece: At undersøge for og reagere på nye symptomer /  Participant handout: Checking and responding to symptoms | Hele pjecen er slettet. | All text/information has been deleted |
| **Andre indholdsændringer /**  Other content changes  Mål / Goals | ***Note S3****. Det består af spørgsmål, så vi kan give dig personlig feedback. Når du har svaret på det første sæt spørgsmål, skal du klikke på "Fortsæt" for at få din personlige feedback og få adgang til det næste sæt spørgsmål****.*** *Når du ser din feedback, vil du også kunne se ”udført”-knappen.* *Du skal klikke på "Udført" for at vise, du er færdig med opgaven.*  Er ændret til:  ***Note S2.*** *Du vil møde de samme spørgeskemaer som i modul 2. Det har vi valgt for at se, om du har ændret opfattelse omkring din bekymring. Når du har svaret på det første sæt spørgsmål, skal du klikke på "Fortsæt" for at sende det til din psykolog. Du skal klikke på "Udført" for at vise, du er færdig med spørgeskemaerne.* | ***Note S3****. It consists of questions so we can provide personalised feedback for you. Once you answer the first set of questions, please click on the “Continue” button to receive your personalised feedback and access the next set of questions. Once you view the feedback, you will also see the “Done” button. Please click on the “Done” button to signify your completion of the task.*  Has been changed to:  ***Note S2.*** *You will meet the same questionnaires as in module 2. We have chosen to do so, to see whether you have changed your perception of worrying. Once you answer the first set of questions, please click on the “Continue” button to send your answers to your psychologist and to receive your personalised feedback. Please click on the “Done” button to signify your completion of the task.* |
| *Side 2:*  */*  *Page 2:* | Al original tekst er blevet genskrevet og skrevet sammen med indholdet af side 4.  *Side 2 – Selvmonitorering og reaktion på nye symptomer*  *Informationen nedenfor har til formål at fortælle om symptomer og tegn på tilbagefald, samt hvad du selv kan være opmærksom på. Der vil også fremgå nogle råd vedrørende at leve godt med stomi.*  *Lægen, der behandlede dig for kræft, er den bedste at spørge om, hvilke symptomer du skal være opmærksom på, og hvor sandsynlige de kan være. Sørg for at fortælle din læge, hvor meget information du gerne vil have. Nogle mennesker vil have hver eneste lille information med det samme, mens andre foretrækker at tage tingene, som de kommer. At udvikle nye symptomer betyder ikke nødvendigvis, at kræften er vendt tilbage. Mange symptomer har intet at gøre med kræft.*  *Følgende symptomer kan være tegn på noget alvorligt, hvis du oplever symptomet i svær grad, eller hvis symptomet er der i flere uger. I det tilfælde anbefales det, at du kontakter din læge.*   - *Blod – med eller uden slim – i afføringen, sort afføring eller ændret afføringsmønster, der varer i mere end to uger.* - *En fornemmelse af, at tarmen ikke tømmes ordentligt, når du er på toilettet* - *Smerter ved afføring* - *Luft i tarmene eller ondt i maven igennem mere end to uger* - *Blodmangel, vægttab eller feber i lang tid uden grund* - *Nedsat appetit, kvalme og eventuelt opkastning* - *Træthed* - *Åndenød* - *Smerter i lænden* - *Eventuelt gulsot (gulfarvning af hud og øjne)* - *Eventuelt kontroltab af blære- og endetarmslukning* - *Følelsesforstyrrelser og lammelser i benene*   *Kilde: Kræftens Bekæmpelse, 2020: Tilbagefald af tyktarmskræft.*  *En hovedregel er – hvis et symptom varer længere end en uge, eller hvis det er meget alvorligt, er det bedst at lave en aftale med din læge.*  *Du kan bruge de færdigheder, du allerede har lært (fx frakoblet opmærksomhed eller udskydelse af bekymring) for at undgå at blive for opslugt af dine bekymringer, mens du venter på, at milde symptomer går væk.*  *Du kan også* [*Klik her*](https://efter.internetbehandling.dk/node/916)*for at downloade information om "’Anbefaling til hvordan du reagerer på symptomer ".*  ***Stomi***  *Mange, der er opereret for tarmkræft, får stomi. Stomi kan tage tid at vænne sig til, men lige så langsomt, vil det normaliseres for dig. Mange er bange for, at andre vil kunne lugte deres stomi. I dag er alle stomiposer udstyret med et kulfilter, og de er derfor lugtfri. Der er forskellige stomiposer, og du kan finde den, der passer til dig. Nogle bekymrer sig om, om deres stomipose kan ses under deres tøj. Med tiden vil du lære, hvordan du bedst går klædt. Prøv at se, om du kan tage en dag ad gangen.*  *Kilde: Kræftens Bekæmpelse, 2020: Stomi ved operation af tyktarmskræft.*  *Klik her, for at downloade pjecen ”Sund livsstil” for mere information om kost og stomi.*  *Du kan få mere information om stomi på Stomiforeningens hjemmeside -* [*https://copa.dk/*](https://copa.dk/)  *Og på Kræftens Bekæmpelses hjemmeside.* | All of the original text has been rewritten and merged with the content of page 4.    *Page 2 – Self monitoring and reaction to new symptoms*  *The information below aims to inform on symptoms and signs recurrence of cancer and what you can be aware of and there will be some advice on how to live well with ostomy.*  *The doctor who provided your cancer treatment is the best person to ask about what symptoms to look out for and how likely they might be. Make sure you let your doctor know how much information you would like. Some people want every last bit of information, while others prefer to just take things as they come.*  *Developing new symptoms does not necessarily mean that the cancer has come back. A lot of symptoms will have nothing to do with cancer.*  *The following symptoms can be a sign of something severe if you experience it extensively or if the symptom last for severe weeks. If so, it is recommended that you contact you doctor.*   - *Blood – with or without mucus – in stool, blac stool or changed bowel habits, lasting more than two weeks* - *A feeling of the intestines not emptying properly when you are on the toilet* - *Pain accompanying stools* - *Intestinal gas or stomach pains lasting more than more than two weeks* - *Long lasting anaemia, weight loss or fever without cause* - *Reduced appetite, nausea and perhaps vomiting* - *Fatigue* - *Shortness of breath* - *Pain across the loins* - *Putatively jaundice (yellow color of skin and eyes)* - *Putatively loss of control of bladder and rectum* - *Sensory disturbances and paralysis in the legs*   *Source: Kræftens Bekæmpelse, 2020: Tilbagefald af tyktarmskræft.*  *As a general rule, if a symptom doesn’t go away in a week, or if it is very severe, it is best to make an appointment in the first instance with your GP.*  *You can use the skills you’ve already learned (e.g. detached mindfulness or worry postponement) to stop you from getting too caught up in your worries while you’re waiting for mild symptoms to resolve.*  *You can also*[*click here*](https://prac-iconquerfear.mydigitalhealth.org.au/wp-content/uploads/2019/02/ConquerFear-Module-4-Handout-2b_180626.pdf%22%20%5Ct%20%22_blank)*to download a handout on "Responding to New Symptoms".*  ***Ostomy***  *Many who receive colorectal cancer surgery will get an ostomy. It can take a while to get used to it, but step by step it will be normalized. Many are afraid that the people around them will smell their ostomy. Because ostomy bags today have a charcoal filter, they are odorless. There is a variety of ostomy bags, and you can find the one that suits you the best. Some people worry that their ostomy bag will be visible under their clothes. With time you will learn how to dress in a way that makes you feel comfortable. Try taking one day at a time.*  *Soruce: Kræftens Bekæmpelse, 2020: Stomi ved operation af tyktarmskræft.*  *Please click here to download the handoutet “healthy lifestyle” for information in diet and ostomy.*  *You can seek more information about ostomy at Stomiforeningens homepage - ttps://copa.dk/*  *And at Kræftens Bekæmpelses home page.* |
| Side 3 / Page 3 | *Spørgsmålene nedenfor skal vise, hvor godt du holder dig til de anbefalede retningslinjer for kontrolundersøgelser. Den vil også komme med anbefalinger vedr. kontrolundersøgelser ift. den type kræft, du har haft.*  *Klik på "Fortsæt" når du har besvaret spørgsmålet nedenfor for at få adgang til flere spørgsmål på denne side. Når du har udfyldt dem, klikker du på "Færdig" for at sende dine svar.*  >>funktion hvor man skulle angive, hvilken form for kræft, man var diagnosticeret med og fik specifik feedback afhængigt af kræftformen <<  ***Bedste praksis***  *Kontrol kan foregå som en kombination af besøg hos din kirurg eller mave-tarm-specialist med sideløbende besøg hos din egen læge eller tilknyttede sygeplejerske. Koloskopi bør udføres 12 mdr. efter operation for, og hvis den første koloskopi var utilstrækkelig, bør den udføres igen 6 mdr. efter operation.*  Er ændret til:  *Nedenfor er anbefalingerne for kontrolforløbet kort angivet. De efterfølgende 2 spørgsmål skal vise, om du følger retningslinjerne for kontrolundersøgelser. Når du har udfyldt spørgsmålene, klikker du på "Færdig" for at sende dine svar.*  ***Gældenden kliniske retningslinjer***  *Opfølgningsforløbet kan omfatte kikkertundersøgelser af tarmen, ultralydsundersøgelse og scanninger afhængig af kræfttype, størrelse og risikofaktorer. Kontrolforløbet er længere, hvis der er givet kemoterapi. Som hovedregel skal de fleste til læge hver 3.-6. måned i 2-3 år efter, at behandlingen er afsluttet. Med tiden bliver kontrolbesøgene mindre hyppige, så efter et par år kan det blive med intervaller på 6 måneder eller årligt i 3-5 år. Opfølgningsforløbet vil være fastlagt af de nationale retningslinjer på området Kirurgisk opfølgning er ofte lidt sjældnere, fordi der ikke har været behov for at give kemoterapi. Ofte vil det være med kikkertundersøgelse efter 1 og 3 eller 5 år.*  >> funktionen til at angive forskellige kræftformer er slettet. Derfor fremgår de kliniske retningslinjer for tyktarmskræft lige efter introduktionsteksten. De efterfølges af de resterende spørgsmål på siden<< | *The survey below checks how well you stick to recommended guidelines on follow-up care. It also provides recommendations on follow-up care in relation to the specific type of cancer you had.*  *After answering the question below, please click on the “Continue” button to access and answer other questions on this page. Once you have completed this, you will see the “Done” button which you should click to submit your responses.*  >>function where you can choose the type of cancer you where diagnosed with and receive specific feedback depending on the type of cancer<<  ***Best pratice information***  *Follow-up can be delivered as a combination of visits to your surgeon or associated gasteroenterologist, with ongoing care by your GP and clinical nurse consultant. Colonoscopy should be performed at 12 months after surgery to exclude missed lesions. If the initial colonoscopy was incomplete then a colonoscopy should be performed at the latest 6 months after surgery****.***  Has been changed to:  *Below the recommendations regarding follow up care is described in short. The following 2 questions will indicate whether you follow the guidelines for follow up care. Once you have completed this, you will see the “Done” button which you should click to send your responses.*  ***Current clinical guidelines***  *Depending of the type of cancer, the size and the risk factors, the follow up care can involve endoscopy of the intestines, ultrasound and scans. The follow-up period is longer if the treatment involved chemotherapy.*  *As a rule of thumb most people have check-ups every 3.-6.th. month in 2-3 years after the treatment has ended. With time the check-up visits get less frequent and after a couple of years with intervals of 6 months or annual in 3-5 years. The follow-up schedule is determined by standard national guidelines for follow-up care.*  *Surgical follow up is often less frequent because chemotherapy has not been necessary. It will often consist of an endoscopy after 1 and 3 or 5 years.*  >> the function that allows you to indicate different types of cancer is removed. Therefor the clinical guidelines appear right after the introduction. It is followed by the remaining questions on the page<< |
| Side 5 (tidligere side 6) / page 5 (former page 6) | *”Overbevisninger”* er ændret til *”tanker”* | *”Beliefs”* are changed to “*thoughts”.* |
| Side 5 (tidligere side 6) / page 5 (former page 6) | Der er i den danske version kun 1 sæt spørgeskemaer. Det er følgende spørgeskemaer:  *Disse spørgsmål vedrører tanker og forestillinger, som man kan gøre sig angående ens egen måde at tænke på. Nedenfor er der en række overbevisninger, folk tidligere har udtrykt. Læs hvert enkelt udsagn og noter, hvor er enig/uenig du er. Besvar alle spørgsmålene – der er ingen rigtige eller forkerte svar.*   - *Min bekymringstendens fortsætter, uanset hvordan jeg prøver at stoppe den* - *Hvis jeg først begynder at bekymre mig, kan jeg ikke stoppe igen* - *Jeg kunne gøre mig selv syg ved at bekymring mig* - *Jeg kan ikke ignorere min tendens til at bekymre mig* - *Min bekymringstendens kunne gøre mig vanvittig* - *Det er farligt for mig, at jeg bekymrer mig* | There will only be 1 sets of questios in the Danish program. It will be the following questionnaires:  *This survey is concerned with beliefs people have about their thinking. Below, there’s a string of beliefs other people have expressed. Read each statement and note how much you agree. Answer all the questions - there are no right or wrong answers.*   \| - *My worrying is dangerous for me* \|  \|  \|  \|  \| \| --- \| --- \| --- \| --- \| --- \| \| - *I could make myself sick with worrying* \|  \|  \|  \|  \| \| - *My worrying thoughts persist, no matter how I try to stop them* \|  \|  \|  \|  \| \| - *I cannot ignore my worrying thoughts* \|  \|  \|  \|  \| \| - *My worrying could make me go mad* \|  \|  \|  \|  \| \| - *When I start worrying, I cannot stop* \|  \|  \|  \|  \| |
| Pjece: Guidelines for Checking your Symptoms Sensibly / Checking and Responding to Symptoms  /  Participant handout:  Checking your Symptoms Sensibly / Checking and Responding to Symptoms | De to pjecer 4 er flettet sammen til ét, og målrettet direkte til tarmkræft. Der er anvendt de alarmsymptomer, som the Danish Cancer Society refererer til på deres hjemmeside. Indholdet i teksten er ikke ændret | The two handouts have been merged and directed specifically to colorectal cancer. It contains the alarm symptoms referred to at The Danish Cancer Society’s webpage. The content of the text isn’t changed. |
| Pjece: opfølgende behandling /  Participant handout:  follow up care | *Som hovedregel skal de fleste til læge hver 3.-4. måned i 2-3 år efter, at behandlingen er afsluttet. Med tiden bliver kontroller gradvist mindre hyppige, så efter et par år kan det blive med 6 måneders eller årlige intervaller i 5-10 år. Hyppigheden af ​​kontrolbesøg afhænger af kræftformen og behandlingen, og for nogle former for kræft afhænger denne tidsplan af de nationale retningslinjer for opfølgende behandling på området. Din kræftlæge og Kræftlinjen (80 30 10 30) kan informere om dette.*  Er ændret til  *Opfølgningsforløbet kan omfatte kikkertundersøgelser af tarmen, ultralydsundersøgelse og scanninger afhængig af kræfttype, størrelse og risikofaktorer. Kontrolforløbet er længere, hvis der er givet kemoterapi.*  *Som hovedregel skal de fleste til læge hver 3.-6. måned i 2-3 år efter, at behandlingen er afsluttet. Med tiden bliver kontrolbesøgene mindre hyppige, så efter et par år kan det blive med intervaller på 6 måneder eller årligt i 3-5 år. Opfølgningsforløbet vil være fastlagt af de nationale retningslinjer på området*  *Kirurgisk opfølgning er ofte lidt sjældnere, fordi der ikke har været behov for at give kemoterapi. Ofte vil det være med kikkertundersøgelse efter 1 og 3 eller 5 år.* | *As a general rule, most people will see their doctor every three to four months for two to three years after their treatment finishes. As time goes on, check-ups gradually become less frequent, so that after a couple of years, checks may be at six-monthly or yearly intervals until reaching five or 10 years. The frequency of follow-up visits depends on the type of cancer and treatment, and for some forms of cancer (for example, breast and bowel cancer) this schedule is determined by standard national guidelines for follow-up care. Your oncologist and the Cancer Council Helpline (13 11 20) can provide information on these.*  Has been changed to:  *Depending of the type of cancer, the size and the risk factors, the follow up care can involve endoscopy of the intestines, ultrasound and scans. The follow-up period is longer if the treatment involved chemotherapy.*  *As a rule of thumb most people have check-ups every 3.-6.th. month in 2-3 years after the treatment has ended. With time the check-up visits get less frequent and after a couple of years with intervals of 6 months or annual in 3-5 years. The follow-up schedule is determined by standard national guidelines for follow-up care.*  *Surgical follow up is often less frequent because chemotherapy has not been necessary. It will often consist of an endoscopy after 1 and 3 or 5 years.* |
| Pjece: opfølgende behandling  Afsnit: indledning  /  Participant handout: follow up care  Section: introduction | *Den opfølgende behandling kan være forvirrende for mange mennesker. Hvad den opfølgende behandling indebærer og begrundelsen for den, kan ofte være uklart for den enkelte. Nedenfor er der nogle oplysninger om opfølgende behandling og nogle eksempler på spørgsmål, som du måske kunne tænke dig at stille din læge som en hjælp til at få mest muligt ud af dine opfølgende lægebesøg.*  Er ændret til  *Det kan være lidt uoverskueligt at få et samlet overblik over opfølgningsforløbet. Nedenfor er der nogle generelle oplysninger omkring opfølgning og nogle eksempler på spørgsmål, som du måske kunne tænke dig at stille din læge som en hjælp til at få mest muligt ud af dine opfølgende lægebesøg* | *Follow-up care can be confusing for many people. What follow-up care entails and the rationale for why it occurs is often poorly understood. We have included some information below about follow-up care and some suggested questions you might wish to ask your doctor to help you get the most out of your follow-up appointments.*  Has been changed to:  *It can be a bit confusing to get an overview of the follow-up care. Below we have included some information on follow-up care and some suggestions for questions you might want to ask your doctor to help you get the most out of your follow-up appointments.* |
| Pjece: Sund livsstil (tidligere sundkost)  /  Participant handout: healthy eating (healthy lifestyle) | Handoutets titel er ændret fra ’sund kost’ til ’sund livsstil’. | The title of the handout ‘healthy eating’ had been changed to ‘healthy lifestyle’. |
| Pjece: Sund livsstil  Afsnit: rygning/  Participant handout: healthy eating (healthy lifestyle)  Section: smoking | *Mere end 10,000 australiere diagnosticeres årligt med kræft relateret til rygning.*  *Lungekræft er den mest udbredte rygerelaterede sygdom, men rygning er også tæt forbundet med kræft i blæren, nyrerne, munden, maven og spiserøret.*  *Stoplinjen vil sende et gratis stop-sæt. Trænede Stoplinje-rågivere kan hjælpe med praktiske råd og ekspertråd, som også kan findes på hjemmesiden:* [*www.quit.org.au*](http://www.quit.org.au)  *De fleste, der prøver at stoppe med at ryge, lykkes ikke i første omgang, så det er vigtigt ikke at miste modet, men snarere se sådan på det, at hvert forsøg er en prøve forud for fremtidig succes.*  Er ændret til:  *Der er evidens for at rygning øger risikoen for tarmkræft. En metanalyse (dvs. en sammenlignende analyse af eksisterende undersøgelser på området) af 28 studier, har vist en forøget risiko for tarmkræft på 20 % for rygerne i sammenligning med ikke-rygere. Sammenhængen mellem passiv rygning og tarmkræft er omdiskuteret, med det tyder dog på, at der er en relativt forhøjet risiko forbundet hermed. Det estimeres at optil 1 ud af 5 af tilfældene af tarmkræft i USA skyldes cigaretrygning. I England estimeres dette tal til 8 %.*  *Det er vigtigt ikke at miste modet, selvom du ikke lykkes med dit rygestop i første omgang. Se snarere sådan på det, at hvert forsøg er en prøve forud for fremtidig succes. Du kan finde information om rygestopstilbud i din kommune hos Stoplinien på deres hjemmeside* [*https://stoplinien.dk/*](https://stoplinien.dk/) *eller ringe til 80 31 31 31* | *More than 10,000 Australians are diagnosed with a smoking-related cancer each year.*  *Lung cancer is the most common, but smoking is also closely linked to cancer of the bladder, kidney, mouth, stomach and oesophagus.*  *The Quitline (13 7848) will mail a free Quit pack. Trained Quitline advisers can help with practical and expert advice, which can also be obtained on the website:* [*www.quit.org.au*](http://www.quit.org.au)  *Most people who try to quit smoking don’t succeed on their first attempt so it is important not to feel daunted, rather viewing each attempt to quit as a rehearsal for future success.*  Has been changed to  *There is evidence thar smoking increases the risk of colorectal cancer. A meta-analysis (an analysis comparing existing studies on the subject) of 28 studies has shown that smokers have 20 % higher risk of colorectal cancer than non-smokers. The relation between secondhand smoking and colorectal cancer is much discussed but there seems to be a relatively increased risk in relation to passive smoking. It is estimated that as much as 1 out of 5 cases of colorectal cancer in USA is caused by cigarette smoking. In the UK the number is estimated to be 8%.*  *It is important not to lose courage even though you don’t have success on your first attempt to quit smoking. You should rather try to view each attempt to quit as a rehearsal for future success. You can find information on the smoking cessation offers in your municipal at Stoplinien at their webpage* [*https://stoplinien.dk/*](https://stoplinien.dk/) *or call 80 31 31 31* |
| Pjece: Sund livsstil  Afsnit: alkohol/  Participant handout: healthy eating (healthy lifestyle)  Section: alcohol | *Alkohol øger sandsynligvis risikoen for tarmkræft hos kvinder samt leverkræft. I modsætning til hjerte-kar-sygdomme er der intet bevis for, at alkohol på noget niveau har nogen beskyttende virkning mod kræft.*  Er ændret til:  *De fleste studier viser, at alkohol er sammenhængende med øget risiko for tarmkræft. Der er intet bevis for, at alkohol har nogen beskyttende virkning mod kræft overhovedet.* | *Alcohol probably increases the risk of colorectal cancer in women and liver cancer. Unlike cardiovascular disease, there is no evidence that alcohol at any level has any protective effect against cancer.*  Has been changed to:  *Most studies show that alcohol is associated with increased risk of colorectal cancer. There is no evidence that alcohol at any level has any protective effect against cancer.* |
| Pjece: Overlevelsesstatistikker – hvad betyder de?  Afsnit: overlevelsesstatistikker – hvad betyder de? /  Participant handout: Making sense of survival statistics  Section: making sense of survival statistics | *For eksempel vil cirka 83 ud af hver 100 mænd (83%), der er diagnosticeret med prostatakræft, være i live fem år efter, at de er diagnosticeret.*  Er ændret til:  *For eksempel viser statistikken fra 2012-2016 at cirka 86 ud af 100 mænd (86%), der diagnosticeres med tarmkræft, være i live 1 år efter diagnosen stilles og 64 ud af 100 (64%) efter 5 år. For kvinder vil 86 ud af 100 (86%), være i live 1 år efter diagnosen stilles, og 67 ud af 100 (67%) efter 5 år.* | *For example, about 83 out of every 100 men (83%) diagnosed with prostate cancer will be alive five years after they are diagnosed.*  Has been changed to:  *For example, the statistic from 2012-2016 shows that about 86 out of every 100 men (86%) diagnosed with colorectal cancer will be alive 1 year after they are diagnosed and 64 out of every 100 (64%) 5 years after. For women 86 out of every 100 (86%) will be alive 1 year after they are diagnosed and 67 out of every 100 (67%) will be alive 5 years after.* |
| Pjece: fysisk aktivitet. Afsnit: At have det godt gennem fysisk aktivitet. /  Participant handout: physical activity.  Section: keeping well through physical activity | ***Forskning har vist, at der er en stor sammenhæng mellem kræft og fysisk aktivitet****. Fysisk aktivitet efter en kræftdiagnose har mange fordele, herunder forbedring af livskvalitet, immunforsvar, kropsopfattelse og mindre træthed, færre smerter og mindre depression, angst og stress.* ***Regelmæssig fysisk aktivitet hjælper også med at holde vægten, og det kan nedsætte risikoen for, at kræften kommer tilbage.*** *Derudover har fysisk aktivitet mange andre sundhedsmæssige fordele, såsom at nedsætte risikoen for at udvikle andre kræftformer og andre sundhedsmæssige problemer som hjerte-kar-sygdomme og sukkersyge og hjælpe de fleste med at se bedre ud og føle sig bedre tilpas! De fleste mennesker synes, at det er meget lettere at på lang sigt at blive ved med at lave aktiviteter, de godt kan lide. Det er vigtigt at vælge aktiviteter, der kan passe ind i en normal dagligdag, og det er godt at tænke på bevægelse som en mulighed og ikke som noget, der er besværligt.*  Er ændret til:   ***Forskning har vist at der er en sammenhængen mellem fysisk aktivitet og nedsat risiko for tarmkræft. Selv moderat fysisk aktivitet er vist sammenhængende med nedsat risiko.*** *Fysisk aktivitet efter en kræftdiagnose har mange fordele, herunder forbedring af livskvalitet, immunforsvar, kropsopfattelse,* ***kondition*** *og mindre træthed, færre smerter* ***og generelt bedre mentalt helbred med mindre angst, stress og depression****. Derudover har fysisk aktivitet mange andre sundhedsmæssige fordele, såsom at nedsætte risikoen for hjerte-kar-sygdomme og sukkersyge, samt at hjælpe de fleste med at føle sig bedre tilpas.* ***Lidt motion er bedre end ingenting og du kan sagtens begynde at motionere, selvom du ikke har motioneret inden du fik kræft.*** *De fleste mennesker synes, det er meget lettere på lang sigt at blive ved med at lave aktiviteter, hvis det er aktiviteter, de godt kan lide. Det er vigtigt at vælge aktiviteter, der kan passe ind i en normal dagligdag, og det er godt at tænke på bevægelse som en mulighed og ikke som noget, der er besværligt.* ***Du må som udgangspunkt motionere så meget du kan og vil, og det anbefales at træningsplaner er individuelt tilrettelagte. Det er vanskeligt at give retningslinjer for træningsformen, men det er vist at progressiv konditionstræning med fordel kan kombineres med styrketræning.*** | ***Research has shown that there is a strong link between cancer and physical activity****. Physical activity after a diagnosis of cancer has many benefits, including improvements in quality of life, immune function, body image and lower levels of fatigue, pain, depression, anxiety and stress.* ***Regular physical activity also helps with weight control; and this may reduce the risk of cancer coming back.*** *In addition, physical activity has many other health benefits, such as reducing the risk of developing other cancers and other health problems like cardiovascular disease and diabetes and helping most people to look and feel better! Most people find that activities they enjoy are much easier to continue long-term. It is important to choose activities that can fit into normal routine, and it is helpful to think of movement as an opportunity, not an inconvenience*  Has been changed to:  ***Research has shown that there is a link between physical activity and reduced risk of colorectal cancer. Even moderate physical has been linked to reduced risk.*** *Physical activity after a diagnosis of cancer has many benefits, including improvements in quality of life, immune function, body image,* ***fitness****, lower levels of fatigue and pain* ***and generally better mental health reducing*** *depression, anxiety and stress.* *In addition, physical activity has many other health benefits, such as reducing the risk of developing other types cancers and other health problems like cardiovascular disease and diabetes and helping most people to look and feel better.* ***A little physical activity is better than nothing and it is no problem to start working out even though you didn’t work out before you had cancer.***  *Most people find that activities they enjoy are much easier to continue long-term. It is important to choose activities that can fit into normal routine, and it is helpful to think of movement as an opportunity, not an inconvenience.* ***I general you are allowed to work out as much as you can and will and it is recommended that training schedules are design specifically to the individual. It is difficult to make guidelines on the type of physical activity, but it is shown that it is profitable to combine progressive cardio training with strength training.*** |

# Modul 5 / module 5

| **Type af ændring** | **Dansk beskrivelse af ændringen** | **English description of the change** |
| --- | --- | --- |
| **Tilføjelse af tekst/information**  Addition og tekst/information  Side 2 / page 2 | *Klik på "Gem" nedenfor* ***for at sende din besvarelse til din psykolog.*** | *Please click on “save” below* ***to send your answers to your psychologist*** |
| Når du har gennemført LivEFTERkræft – hvad så? /  After completing iConquerFear – what next? | *LivEFTERkræft er et nyt behandlingskoncept, og du har deltaget i et klinisk lodtrækningsforsøg, hvor halvdelen fik tilbudt LivEFTERkræft, og den anden halvdel fungerede som kontrolgruppe. For at vurdere effekten af LivEFTERkræft udsender vi opfølgningsspørgeskemaer til din e-Boks om få dage, og om 3, 6 og 12 måneder.*  *Vi håber, at LivEFTERkræft er så effektivt, at vi kan argumentere for, at det skal indføres som et blivende behandlingstilbud til mennesker, der har haft kræft, og det er derfor rigtig vigtigt, at du besvarer de spørgeskemaer, vi sender ud. Tak.*    *Alle os bag dette studie vil gerne sige tak for den energi og tid, du har brugt på at arbejde med din frygt for tilbagefald af kræft. Vi håber virkelig, at du er blevet hjulpet, og at denne behandling har givet dig redskaber til, at du kan håndtere din frygt på en ny måde, som gør, at du kan stræbe imod de værdier og mål i livet, som er vigtige for dig, og kan leve et godt og meningsfuldt liv trods det kræftforløb, du har været igennem.  Vi ønsker dig held og lykke fremover.* | *LivEFTERkræft is a new treatment concept and you have participated in a randomized controlled clinical trial*  *where half of the participants were offered LivEFTERkræft and the other half functioned as a control group. We will send a follow-up questionnaire to your e-boks in a few days and in 3, 6 and 12 months, in order to assess the effect of LivEFTERkræft.*  *We hope that LivEFTERkræft will be so effective, that we can make a case for it to become a permanent treatment option to people who have had cancer and that is why it is of great importance that you answer the questionnaires we send. Thank you.*  *Everyone behind this study would like to thank you for the energy and time, you have used working with your fear of cancer coming back. We sincerely hope that you have been helped and that this treatment has given you tools enabling you to handle your fear in a new way that makes you able to strive for the values and goals in life important to you to live a good and meaningful life in spite of the cancer course you have gone through.*  *We wish you the best of luck in the future.* |
| **Andre indholdsændringer**  Other content changes  Side 4  Afsnit: min nye plan /  Page 4  Section: my new plan | ***Adfærd***  *"Jeg vil undgå gentagne gange at undersøge mine bryster udover de sædvanlige månedlige selv-undersøgelser."*  *"Jeg vil undgå at bruge lang tid foran spejlet for at se på min hud og kun grundigt tjekke min hud én gang om måneden."*  Er ændret til:  ***Adfærd*** *”Jeg vil besøge steder jeg kender, også hvis de minder mig om kræft”*  *”Jeg vil tale med mine venner og familie om kræft, hvis jeg får lyst eller har behov for det.” ”jeg vil overholde mine aftaler med min læge”* | ***Behavior***  *"I will avoid repeatedly re-examining my breasts outside of my usual monthly breast self-examination times."*    *"I will avoid spending prolonged periods in front of the bathroom mirror looking at my skin and only thoroughly check my skin for signs of recurrence once a month."*  Has been changed to:  ***Behavior***  *“I will visit places I know also if they remind me of cancer”*  *“I will take to my friend and family about cancer I feel like it or need it”*  *“I will keep my appointments with my doctor”* |
| Side 5 / page 5 | *’aktiviteter’* er kaldet *’råd’* i stedet for. | *’activities’* is called *’advise’* instead. |
| Side 5 / page 5 | *Flot at du er kommet til dette trin i modul 5, og at du har gennemført alle moduler i programmet. Der er et par ting, du skal være opmærksom på, når du afslutter program.*  Er ændret til  *Flot klaret. Du har nu gennemført alle modulerne i programmet og er ved vejs ende. Her er et par råd til dig - til dit videre arbejde med at håndtere frygten for tilbagefald.* | *Well done for getting to this stage of Module 5 and for completing all the modules in this program. There are a few things to be noted here as you finalise this program.*  Has been changed to:  *Well done. You have now completed all the modules in the program and have come to the end of the process.*  *Here is some advice for you – to your further work with handling the fear of cancer returning.* |
| Side 5 / page 5 | *Aktivitet 2: fortsæt med at bruge de redskaber, du har lært i LivEFTERkræft.*  *Fortsæt med at bruge de færdigheder, du har lært i iConquerFear som reaktion på enhver form for frygt for, at kræften kommer tilbage. Nogle af de færdigheder du har lært er; Løsrevet mindfulness, Bekymringsudskydelse og Udfordring af ugunstige overbevisninger.*  *Du kan klikke her for at gå til ‘Redskaber-på-farten", hvor du nemt kan få adgang til disse ressourcer på én gang.*  *Aktivitet 3: Brug din "Nye plan" til at håndtere din frygt for tilbagevendende kræft*  *Hvis du oplever et tilbagefald i adfærd, gå da tilbage til den “Nye plan”, du lavede tidligere i dette modul for at hjælpe dig til at håndtere din frygt for tilbagevendende kræft. Klik her for at få adgang til “Ny plan”.*  Er ændret til:  *2. råd: fortsæt med at bruge de redskaber, du har lært i LivEFTERkræft.*  *Husk, at det ikke er en fejl, når du oplever frygt for tilbagefald af kræften igen. Det er fortsat en normal reaktion efter en kræftdiagnose og -behandling, og ikke noget du slipper helt af med. Det vigtige er, hvordan du håndterer din frygt.  Fortsæt med at bruge de redskaber, du har lært i LivEFTERkræft som reaktion på enhver form for frygt for tilbagefald af kræft. Du kan klikke her for at gå til ”Redskaber", hvor du nemt kan få adgang til alle ressourcer på én gang.  Det tager tid at ændre vaner og tankemønstre, så hav tålmodighed med dig selv.*  *3. råd: Brug din "Nye plan" til at håndtere din frygt for tilbagefald af kræft*  *Hvis du oplever et tilbageskridt i din evne til at tackle frygten for tilbagefald af kræft, gå da tilbage til den “Nye plan”, du lavede tidligere i dette modul, for komme tilbage på sporet. Hold fokus på dine værdier, og styr i retning af dem i stedet for at forsøge at styre uden om frygten.*[Klik her](https://efter.internetbehandling.dk/node/911)*for at få adgang til din “Nye plan”.* | *Activity 2: Continue use of skills learnt on LivEFTERkræft* *Continue to use the skills you have learnt on LivEFTERkræft in response to any other fears of cancer returning. Some of the skills you have learnt include; Detached Mindfulness, Worry Postponement, and Challenging Unhelpful Beliefs.*  *Consider*[*clicking here*](https://prac-iconquerfear.mydigitalhealth.org.au/27318-2/)*to access the ‘on the go tools’ page where you can easily access these resources at once.*  *Activity 3: Use your "New Plan" to deal with fears of cancer returning*  *Return to the “New Plan” you earlier created in this module to help you deal with fears of cancer returning if you experience a relapse. Consider*[clicking here](https://prac-iconquerfear.mydigitalhealth.org.au/module-5-page-5-developing-new-plans/)*to access the “New Plan”.*  Has been changed to:  *2. advice: Continue use of skills learnt on LivEFTERkræft*  *Remember that it is not a mistake, when you again experience fear of cancer returning. It is still a normal reaction after a cancer diagnose and treatment and isnøt something you will get rid of completely. The important thing is how you handle your fear.*  *Continue to use the skills you have learnt on LivEFTERkræft in response to any other fears of cancer returning. You can click here to go to ‘Tools on the go’ where you easily get access to all the resources at once.*  *It takes time to change habits and thought patterns so be patient with yourself.*  *3. advice: Use your "New Plan" to deal with fears of cancer returning*  *Return to the “New Plan” you earlier created in this module to help you deal with fears of cancer returning and get back on track if you experience a relapse. Stay focused on your values and aim towards them instead of trying to steer clear of the fear. Click here to access your “New Plan”.* |
| Når du har gennemført LivEFTERkræft – hvad så? /  After completing iConquerFear – what next? | *2. Du kan altid vende tilbage til iConquerFear for at få frisket værktøjer og strategier op. Klik her for at gå direkte til værktøjerne.* Er ændret til:  *Det næste halve år har du fortsat adgang til LivEFTERkræft, hvor du kan få frisket redskaber og strategier op. Klik her* *for at gå direkte til redskaberne.*  *Din psykolog er tilknyttet de 10 uger, du er i aktiv behandling. Hvis du har brug for yderligere psykologhjælp, kan du få en henvisning via din egen læge. Beskederne ligger der stadig, så du kan genlæse dem, hvis du har lyst.*  *Du må gerne downloade alle pjecerne i LivEFTERkræft til dig og din familie.*  *Klik her for at gå til pjecerne.* | *2. You can always come back to iConquerFear to brush up on tools and strategies you have found out about. Please*[*click here*](https://prac-iconquerfear.mydigitalhealth.org.au/27318-2/)*to access those tools directly.*  Has been changed to:  *You will have access to LifEFTERkræft the next six months allowing you to brush up on the tools and strategies.*  *Click here to get directly to the tools.*  *Your psychologist is connected the 10 weeks, you are in active treatment. If you need further psychological counselling, you can get a referral from your general practitioner. The messages will remain in order for you reread them if you want to.*  *You can download all the handouts in LivEFTERkræft for yourself and your family.*  *Click here to access to the handouts.* |

New handouts:

# Long-term side effect

**Physical and psychosocial long-term side effects of cancer**

Changes that appear as a consequence of cancer disease and cancer treatment is called long-term side effects. Long-term side effects are mental, physical and psychosocial decreased functionality that are debilitating to everyday life. Sundhedsstyrrelsen (The Danish Health Authority) define long-term side effects as: *health issues that arise during primary treatment and become chronic or that arise and manifest months or years after the treatment has ended. Long-term side effects comprise new primary cancer disease and physical, mental or social changes as a result of cancer disease and/or the treatment of the disease.*

To more than every other cancer patient in Denmark long-term side effects are a problem in their everyday life. Cancer in the intestines, the urinary tract and the abdomen organs is followed by pains, incontinence, sexual problems, constipation and diarrhea. Many cancer patients feel that the physical and mental long-term side effects affect the social and practical aspects of their everyday life as for example returning to work after a cancer treatment procedure. Below you can read more about common long-term side effects that other cancer survivors experience.

***Changed body image***

Changed body image or body perception after cancer treatment is a frequently reported long-term side effect. It can be accompanied with loss of identity and it can have a negative effect on quality of life.

There are multiple exercises for restoring confidence and familiarity with your body after cancer treatment. For example, through zone therapy, music therapy, sensuality training and physical activity. Some benefit from other kinds of exercises such as looking in the mirror or creating sensory experiences.

You can read more about getting to know your body at Kræftens Bekæmpelses home page: <https://www.cancer.dk/hjaelp-viden/hvis-du-har-kraeft/seksualitet/det-kan-du-selv-goere/laer-din-krop-at-kende/>

***Sexual problems***

As much as half of all cancer patients have sexual long-term side effects. There are many factors that can affect and cause these sexual problems, and they can be both mental and physical. The physical long-term side effects are dry mucous membranes, injured nerves, infections, incontinence and pains. Among the mental challenges people experience are negative body image, anxiety and depression. Antihormonal treatment can entail a reduced or an absent sexual desire, and for men it can also include erectile dysfunction.

Because of the bodily changes that cancer treatment can cause, you might have to rediscover your relation to your body, and it might cause you to feel less sexually attractive. Some people feel shy or ashamed when they are naked if cancer treatment has changed their body. If you have a hard time feeling safe with your body, it can be difficult to relax and believe that other like your body and find it attractive. After cancer treatment it can be important to work with body acceptance and start connecting your body with something joyful. One way to do this is by creating positive bodily experiences, for example through a massage. You can read about this in the section “changed body image”. You can also learn about sensuality training at Kræftens Bekæmpelses home page: <https://www.cancer.dk/hjaelp-viden/hvis-du-har-kraeft/seksualitet/det-kan-du-selv-goere/sensualitetstraening/>

Some people find it relieving to talk to their partner about sexual worries, emotions and expectancies. You can find inspiration on how to talk about sexuality here: <https://www.cancer.dk/hjaelp-viden/hvis-du-har-kraeft/seksualitet/samliv/inspiration-samtalen/>

Some men experience erectile dysfunction after cancer treatment. There are different treatment options depending on the cause of the issue and therefor it can be a good idea to talk to your doctor about the type of treatment that is most suitable to you. Even though you can’t get an erection or complete an intercourse many can still get an orgasm by stimulation with hand, mouth or a vibrator. Sex is many things besides intercourse. You can read more about other ways of having sex besides intercourse here: <https://www.cancer.dk/hjaelp-viden/hvis-du-har-kraeft/seksualitet/det-kan-du-selv-goere/sex-paa-mange-maader/>

Sensitive or dry mucous membranes can give rise to arching and irritation around the labia, clitoris and vagina. There are multiple treatment options that you doctor can inform you of. If you experience pains deep in the vagina during intercourse it can be due to adhesion after your surgery or due to radiotherapy. But it can also be caused by tight and sore muscles and ligaments in the abdomen area, at which case a specialized physiotherapist often will be able to relieve it.

It is common to need help with these kinds of problems. According to a survey by Kræftens Bekæmpelse from 2013 more than every third cancer patient needs help handling sexual problems. Some benefit from talking to a sexologist, psychologist or doctor.

***Social problems***

For cancer survivors, social relationships can be heavily loaded. Persistent anxiety and depression symptoms, issues regarding social roles, unfulfilling support or communication issues can contribute to poor relations. The dependents adaption can also be affected by busy or sensitive periods in their lives.

***Tiredness / fatigue***

Cancer related fatigue is a major and sudden emotional, cognitive and physical tiredness that isn’t equivalent to the exertion level or reduced by resting. It isn’t the same as the tiredness that often follows a cancer treatment. It is often accompanied by concentration problems, unease, reduced stamina, poor short-term memory, sleep disturbance, physical discomfort, reduced interest in activities and heightened sensitivity to sensory inputs.

Physical activity can also have a positive influence on fatigue. Having fixed everyday routines and planning activities can also help, as well as sitting down doing everyday activities.

Often it will be possible to make an agreement with your employer about working hours and options for rest and assignments.

***Sleep disturbances***

Sleep disturbances cover problems regarding falling to sleep, disturbed sleep, light and short sleep and a feeling of exhaustion during the day. Sleep disturbances often occur together with tiredness, anxiety, pains and depression. In can affect the daily function and quality of life for example by reducing the energy to everyday practicalities, to being with your close family or reducing your concentration. Even though poor sleep can reduce the immune system nothing seems to indicate that sleeplessness affects the chance of surviving cancer.

Chronical sleeplessness is often caused by unfavourable sleep and though patterns. Kræftens Bekæmpelse gives 5 good pieces of advices on sleep:

- Avoid laying wake in your bed. This means that if you’re awake more than 20-30 minutes you should go do something else that relaxes you.
- Keep electronic devices such as TV, tablet, computer and mobile phone out of your bedroom and turn of this kind of screens off an hour before you want to sleep.
- Wake up at the same time every day also during the weekends no matter how many hours you have slept.
- Only go to bed when you feel sleepy.
- Avoid sleeping during the day or early evening. If you can’t stay awake, you can take a nap before 3 pm lasting at the maximum an hour.

Physical activity can also reduce sleep problems.

***Problems regarding the intestines and urinary***

As much as 50 % of all patients who have had colorectal surgery experience troublesome bowel movements, especially if it was combined with radiotherapy. To some the intestinal long-term side effects are so severe that they affect people’s social life.

In the handout “healthy lifestyle” you can read more about dietary recommendations also in relation to ostomy.

***Dumping syndrome***

Dumping syndrome can arise after surgery or removal of the stomach. The symptoms of dumping syndrome are discomfort, nausea, vomiting, bloating, stomach aches, diarrhea, dizziness and tiredness. Some people also experience palpitation and sweats. The symptoms appear 1-3 hours after a meal and often disappear without treatment.

After stomach surgery the food is send directly and faster from the stomach to the intestines and this is what causes dumping syndrome. The fast transition causes the sugar concentration in the intestines to rise and fluid to soak in, so it distends and works faster.

Discomfort and nuisance can often be helped by changing your eating habits. You can read more on dietary recommendations for dumping syndrome in the handout “healthy life-style”.

***Pains***

Many cancer patients suffer from pain especially during the first 5 years after they are diagnosed. Pain can affect the quality of life, mood, sleep and the ability to get back to work and other activities. After a surgery muscular pains and pains in the internal organs can occur. After radiotherapy some people are in pains due to loss of tissue and to scarring. Antihormonal treatment can lead to pain in muscles and joints and chemotherapy can give rise to neuralgia. Especially neuralgia is difficult to treat but there are different treatment options that you doctor can inform you on.

***Anxiety and depression***

Anxiety is an unpleasant and serious long-term side effect of cancer. The same goes for depression. Big questions about the meaning of life will appear both during and after the cancer treatment – and that’s natural. Depression is dominantly experienced in the period right after and up till two years after receiving a cancer diagnose.

It is normal to experience difficult emotions in relation to cancer. These emotions can match the symptoms of depression but will as at starting point be considered as a natural reaction to your situation. If the symptoms are prolonged, you should contact your doctor and let her help you on how to get better. It isn’t the cancer but the strain that comes with the disease that causes a depression. Although only a few will actually experience that their feelings of low spirits and despondency turn into a depression and need treatment it is important to take the feelings serious. Depression is different from the natural reaction to cancer because it is persistent. With a natural reaction you more often have periods with a better mood, for example if your family or friends come to visit or if you receive a positive treatment result. These upswings won’t appear or won’t be as big during a depression.

The typical signs of depression are:

- Low spirits
- Reduced zest for life
- Unexplained and lasting tiredness

Some people also experience:

- Reduced self-worth
- Self-reproach and sense of guilt
- Difficulties concentrating
- Sleep disturbances and changed appetite

How many symptoms you have, how they are expressed and for how long they have been present is decisive to whether you have a depression or not.

In this moment there is no standardized treatment offers to mental long-term side effects after cancer, but it is an area in great progress. If your doctor finds that you have a depression you will be offered to talk to a psychologist maybe combined with anti-depressive medicine. Some practitioners also offer sessions for support and talk. Through the National Health Service, you can get as much as 7 supportive sessions with your doctor free of charge. If you have a referral from your doctor to see a psychologist, you can get a subsidy of 60% of the usual fee. You can get subsidy to maximal 12 session but if you get another referral you can get subsidy for 12 extras. The referral must be given within 12 months from the time of diagnosing.

There are some things you can do to easy your difficult emotions:

- **Allow yourself to have a mental reaction:** being sad once in a while is not the same as being depressed. It is natural for the mood to change. Try not to be too hard on yourself when you have a mental reaction but allow yourself to be low in spirits sometimes. And try to regard your emotional reactions as an expression of the strain that cancer is or has been.
- **Share your thoughts with others:** some find it helpful to talk to others about their thoughts and feelings. It can give you a sense of relief and comfort and it can reduce the feeling of loneliness that many experience. Try to maintain your social activities if you feel it lifts your mood – also if the low spirits might return, when you are alone again.
- **Physical activity:** to be physically active can help reducing anxiety and depression. It doesn’t have to be demanding training. Even a walk can be helpful. To some people exercising is an opportunity to put their mind to something else and to others it is a room to think things through. Find out what works best for you.
- **Write down your thoughts and activities:** as you’ve learned in this programme it is sometimes easier to put away your thoughts if you write them down. You can also try to note down your daily activities. If you review the activities of your day and rate them with a number between 1 (the worst mood) and 10 (the best mood) you can get a general view of what activities elevate your mood.

***Cognitive problems***

Cognitive problems include difficulties in learning, planning, memory and concentration. 15-25 % of all cancer survivors experience cognitive problems after treatment has ended. The issues can arise during chemotherapy, but all types of treatment can be ‘coresponsible’ in causing the problems. Sleep disturbances, depression, tiredness and anxiety can also contribute.

Chemo brain or chemo fog is a state experienced by some cancer patients during chemotherapy, surgery, hormone treatment and radiotherapy. The symptoms of chemo brain are:

- Reduced concentration level.
- Reduced memory
- Difficulties remembering and recalling places, names and messages
- To get easily distracted
- Problems multi-tasking

There is uncertainty to what causes the symptoms and they often vanish without treatment.

Many find that shopping lists, checklist, have fixed routines and GPS’s ease everyday life.

Region Midt has developed a tool sheet that might be helpful to you. You can access the sheet here: : <https://www.cancer.dk/dyn/resources/File/file/4/8754/1591082450/redskabsark-koncentration-og-hukommelse.pdf>.

You can listen to the personal experiences and stories of others and read more about chemo brain at Regions Midts page Liv og kræft here: <https://www.livogkraeft.rm.dk/koncentrationoghukommelse/>

***Lymphoedema***

Lymphoedema describes a chronic swelling and 50-75% of all cases is caused by cancer treatment. Lymphoedema often appears in arms or legs, but it can also break out in the chest, reproductive organs or the head and neck area. Even though lymphoedema is a chronic illness there are good chances of limiting and controlling it and to ensure a minimum of discomfort. The treatment consists of bandaging performed by a specially trained lymph-therapist to remove accumulated lymph-fluids, constant use of compression socks and skin care. If the treatment doesn’t help and it persist causing irritation, then liposuction is an option.

***Senfølgerforeningen (the long-term side effect association)***

At Senfølgerforeningens homepage you can join a network group, ask questions to a welfare officer regarding work and long-term side effects and read about other cancer survivor’s experiences with long-term side effects. You can also contact the telephone hotline and talk to someone who has either personal experiences with long-term side effects or is close to some with long-term side effects of cancer. The number is: 40 44 78 48.
Their home page is: <https://www.cancer.dk/senfoelger/>

***Kræftens Bekæmpelses advisory centers***

Kræftens bekæmpelse has advisory centers throughout the country. At the centers you can get advice as to how you can get the best help at taking care of your long-term side effects.
You can find your local advisory center here: <https://www.cancer.dk/hjaelp-viden/raadgivning/radgivninger/>

***Acknowledgements***

This information contained in this information is adapted from information by Senfølgerforeningen: Har jeg senfølger?. Samt fra Bedre Viden Om Senfølger: Helbredt – men ikke rask?(<https://www.cancer.dk/dyn/resources/File/file/8/7388/1529311425/kb_senfoelger_opslag.pdf>) information developed by Kræftens Bekæmpelse (Lær din krop at kende efter kræftbehandling: <https://www.cancer.dk/hjaelp-viden/hvis-du-har-kraeft/seksualitet/det-kan-du-selv-goere/laer-din-krop-at-kende/>, Seksualitet og ændret udseende: <https://www.cancer.dk/hjaelp-viden/hvis-du-har-kraeft/seksualitet/seksuelle-problemer/hvis-kroppen-aendrer-udseende/>, Seksuelle problemer hos kvinder efter kræftbehandling: <https://www.cancer.dk/hjaelp-viden/hvis-du-har-kraeft/seksualitet/seksuelle-problemer/seksuelle-problemer-kvinder/>, Seksuelle problemer hos mænd efter kræftbehandling: <https://www.cancer.dk/hjaelp-viden/hvis-du-har-kraeft/seksualitet/seksuelle-problemer/hos-maend/>, Det kan du gøre ved træthed: <https://www.cancer.dk/hjaelp-viden/bivirkninger-senfolger/traethed-og-soevnloeshed/det-kan-du-goere-ved-traethed/>, Søvnløshed: <https://www.cancer.dk/hjaelp-viden/bivirkninger-senfolger/traethed-og-soevnloeshed/soevnloeshed/>, Dumping syndrom: <https://www.cancer.dk/hjaelp-viden/bivirkninger-senfolger/dumping-syndrom/>, Kemohjerne: <https://www.cancer.dk/hjaelp-viden/bivirkninger-senfolger/kemohjerne/>, Depression ved kræftsygdom: <https://www.cancer.dk/hjaelp-viden/hvis-du-har-kraeft/psykiske-reaktioner/depression/?gclid=EAIaIQobChMIrPSfytXt6gIVAbp3Ch3X-QvIEAAYASAAEgKhG_D_BwE>, Tilskud til psykologsamtaler: <https://www.cancer.dk/hjaelp-viden/rettigheder/oekonomisk-hjaelp/tilskud-psykologsamtaler/>, Lymfødem: <https://www.cancer.dk/hjaelp-viden/bivirkninger-senfolger/lymfoedem/>, behandling af lymfødem: <https://www.cancer.dk/hjaelp-viden/bivirkninger-senfolger/lymfoedem/behandling/>). Information developed by Sundhedsstyrelsens Vidensopsamling på senfølger efter kræft hos voksne (<https://www.sst.dk/~/media/8D196FE8A2D14B9E838908BB23F288A4.ashx>) and Liv med og efter Kræfts side om psykisk påvirkning (<https://www.livogkraeft.rm.dk/psykiskpaavirkning/>) is also used in developing this handout.

# Screening

**Screening for colorectal cancer**

Every year c. 5.000 Danes are diagnosed with colon and rectal cancer. Every second year all seemingly healthy citizens in the age of 50-74 are offered a screening with the object of detecting colorectal cancer or initial stages of colorectal cancer. Screening helps early detection in initial stages and reduces the risk of dying of colon and rectal cancer.

If 10.000 are screened there will be found signs of blood in 677 of the cases, 179 will have polyps and 37 will have colorectal cancer.

***The screening procedure***

A screening involves a stool sample. You receive a testing kit by mail and collect the stool sample yourself. The sample is sent to the lab where it is examined for signs of blood. You get the test results within 14 days. If the stool sample shows sign of blood you should have an endoscopy. Before the endoscopy you will have to cleanse your intestines with laxative. The examination will show whether there is cancer or polyps that can develop into cancer.

If the endoscopy shows that your colon and rectum is normal your risk of developing cancer in this arear is very low. Therefor you won’t be offered scans for another 8 years if you are younger than 75 years.

***Screening after cancer treatment***

If you have been treated for colorectal cancer you will get an endoscopy at the follow up care to check for new polyps and lumps. If there is nothing to be found, you will have screenings every fifth year until you turn 75. The risk is then limited. If the endoscopy shows a polyp or a lump, it will be surgically removed, and you will be offered check up every third or fifth year depending on the kind of polyp.

1 and 3 years after you have had a radical surgery (i.e. you have had all probable cancer removed) you will get a CT-scan of the abdominal cavity and thoracic cavity to check whether the cancer has spread to other organs. If there is found no sign of cancer, you don’t need additional CT-scans.

**Acknowledgements**

The informaiton in this handout is adapted from information by Kræftens Bekæmpelse (Screening for tarmskræft: <https://www.cancer.dk/forebyg/screening/screening-tarmkraeft/saadan-foregaar-undersogelsen/>, Opfølgning efter tyktarmskræft: <https://www.cancer.dk/tyktarmskraeft-coloncancer/kontrol-tyktarmskraeft/>) og from Sundhedsstyrelsen (Tilbud om undersøgelse for kræft i tyk- og endetarm: <https://www.sst.dk/-/media/Udgivelser/2016/Tarmkr%C3%A6ftscreening/Pjece--Tarmkr%C3%A6ftscreening.ashx>)
