# Supplementary material for: Adapting a Self-Guided eHealth Intervention Into a Tailored Therapist-Guided eHealth Intervention for Survivors of Colorectal Cancer
Source: JMIR Cancer. 2025 Mar 5;11:e63486. doi: 10.2196/63486 (PMC11900901; doi:10.2196/63486)
Supplement: Multimedia Appendix 2 [file cancer-v11-e63486-s002.docx]

**
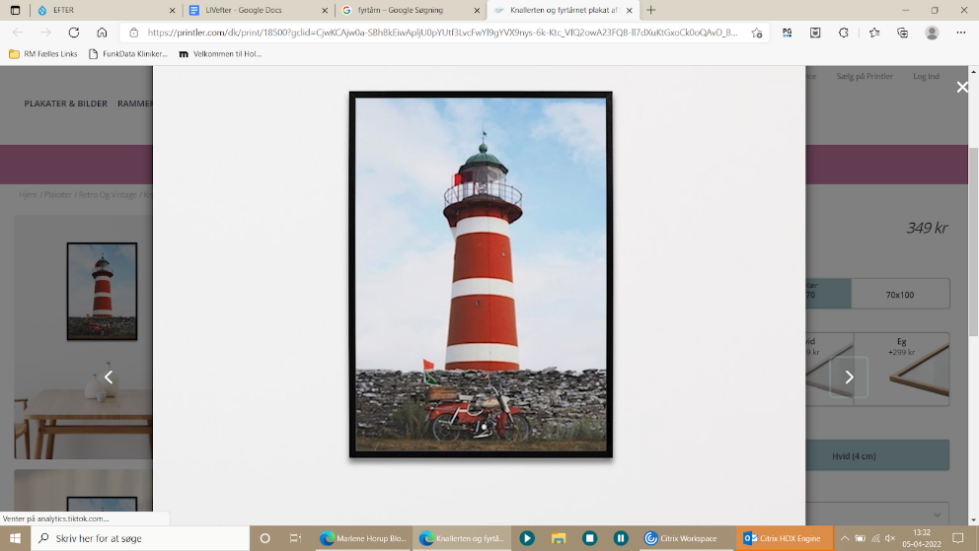
***“Values can act as beacon in our lives. Values can guide us through both good and bad times. In this way, values can give us a form of life direction, because they help us to live a life that is in accordance with what is important to us and to the person we want to be. We can compare the values with a lighthouse: When the ship is sailing out at sea in darkness and storm, the light from the lighthouse can help the ship stay on course and reach port safely. In the same way, values can act as our lighthouse when there is darkness and storm in our lives and in periods when it is difficult to find direction.”*

*Exercise: Life deviation*

*• Now select one of the most important values and insert the value under the item value.*

*• Under importance, give the value a number from 0-10, where 10 is the most possibly importance*

*• Under current behaviour, give a number from 0-10 on how much you live by this value at the moment, i.e. how much you feel your current behavior supports the value.*

*• Now calculate the life deviation by subtracting the current behavior number from the importance number, see example.*

| ***Value*** | ***IMPORTANCE*** | ***CURRENT BEHAVIOUR*** | ***LIFE DEVIATION*** |
| --- | --- | --- | --- |
| *Example: Close relationships* | ***9*** | ***3*** | ***6*** |

*You may already by living according to some of your most important values. Choose one of your most important values with the greatest life deviation, that you want to take care of now. Perhaps a value that is important to you, but due to your fear of cancer recurrence has diminished.*

*Based on the value you must now formulate an action that is in accordance with your chosen value, i.e. set a goal for how you want to work with the value. A goal which is in line with the value and which can be achieved, as opposed to a value which shows direction and which we never reach.*

*Exercise: My value-based goals
Chosen value = close relationships*

*Choose 1-3 small actions (they must be simple and doable within the next 24 hours):*

*Example: Write an invitation to an old friend about an upcoming fishing trip/walk.*

*And*

*Choose 1-2 major actions (something I can do within the next days or week/weeks):*

*1. Arrange a working day*

*2. Have a working day with children and grandchildren*

*At the end:*

*How sure are you that you can achieve your/your goals?*
